# ATR-mediated DNA damage responses underlie aberrant B cell activity in systemic lupus erythematosus

**DOI:** 10.1126/sciadv.abo5840

**Published:** 2022-10-28

**Authors:** Theodora Manolakou, Dionysis Nikolopoulos, Dimitrios Gkikas, Anastasia Filia, Martina Samiotaki, George Stamatakis, Antonis Fanouriakis, Panagiotis Politis, Aggelos Banos, Themis Alissafi, Panayotis Verginis, Dimitrios T. Boumpas

**Affiliations:** ^1^ Center for Clinical, Experimental Surgery and Translational Research, Biomedical Research Foundation of the Academy of Athens, 115 27 Athens, Greece.; ^2^ School of Medicine, National and Kapodistrian University of Athens, 115 27 Athens, Greece.; ^3^ Center for Basic Research, Biomedical Research Foundation of the Academy of Athens, 115 27, Athens, Greece.; ^4^ Institute for Bioinnovation, Biomedical Sciences Research Center Alexander Fleming, Vari, Attica, Greece.; ^5^ Centre of New Biotechnologies and Precision Medicine (CNBPM) School of Medicine, National and Kapodistrian University of Athens, Athens 115 27, Greece.; ^6^ Department of Rheumatology, Asklepieion General Hospital, Athens, Greece.; ^7^ School of Medicine, European University Cyprus, 1516, Nicosia, Cyprus.; ^8^ Laboratory of Biology, National and Kapodistrian University of Athens Medical School, 124 62 Athens, Greece.; ^9^ Institute of Molecular Biology and Biotechnology, Foundation for Research and Technology, 700 13 Heraklion, Greece.; ^10^ Laboratory of Immune Regulation and Tolerance, Division of Basic Sciences, University of Crete Medical School, 700 13 Heraklion, Greece.; ^11^ Joint Rheumatology Program, 4th Department of Internal Medicine, Attikon University Hospital, National and Kapodistrian University of Athens Medical School, 124 62 Athens, Greece.

## Abstract

B cells orchestrate autoimmune responses in patients with systemic lupus erythematosus (SLE), but broad-based B cell–directed therapies show only modest efficacy while blunting humoral immune responses to vaccines and inducing immunosuppression. Development of more effective therapies targeting pathogenic clones is a currently unmet need. Here, we demonstrate enhanced activation of the ATR/Chk1 pathway of the DNA damage response (DDR) in B cells of patients with active SLE disease. Treatment of B cells with type I IFN, a key driver of immunity in SLE, induced expression of ATR via binding of interferon regulatory factor 1 to its gene promoter. Pharmacologic targeting of ATR in B cells, via a specific inhibitor (VE-822), attenuated their immunogenic profile, including proinflammatory cytokine secretion, plasmablast formation, and antibody production. Together, these findings identify the ATR-mediated DDR axis as the orchestrator of the type I IFN–mediated B cell responses in SLE and as a potential novel therapeutic target.

## Teaser

DNA damage response as a critical regulator of autoimmune B cell responses in SLE disease.

## INTRODUCTION

DNA damage response (DDR) mechanisms represent a dynamic feature of immune system development and function. Key roles of DDR include monitoring the genomic rearrangements required by lymphocytes to form antigen receptors, generate antibodies, mature, and divide rapidly to respond to infections (*1*, *2*). Persistent DDR caused by genetic or epigenetic factors is a driving feature for malignant diseases and developmental syndromes. Notably, aberrant DDR has been recently reported in a wide range of autoimmune diseases, including systemic lupus erythematosus (SLE), multiple sclerosis (MS), rheumatoid arthritis (RA), and Aicardi-Goutières syndrome (*1*, *3*–*6*). However, the molecular complexity of DDR pathways, together with the lack of knowledge on the context- and cell-dependent DDR engagement, has hampered the therapeutic targeting of DDR in these diseases.

The main transducers of the DDR signaling are the three protein kinases ATM (ataxia-telangiectasia mutated), ATR (ATM and Rad3 related), and DNA-PKcs (DNA-dependent protein kinase catalytic subunit) (*7*). ATM and DNA-PKcs are considered as the pinnacles of the double-strand DNA breaks signaling cascade, whereas ATR is mainly activated upon single-strand DNA breaks and responds to agents causing DNA replication stress, such as ultraviolet light (*8*, *9*). Upon activation, these DDR components may phosphorylate separately or synergistically a number of proteins including checkpoint kinase 2 (Chk2; Thr^68^, by ATM), Chk1 (Ser^345^, by ATR), H2AX (Ser^139^, γΗ2ΑΧ) and p53 (Ser^15^), determining cellular response and fate (*8*). Notably, p95/NBS1 (also called nibrin) usually colocalizes with γΗ2ΑΧ at the damaged genomic site and may propagate both ATR- and ATM-dependent signaling (*10*, *11*). Although ATM, ATR, and DNA-PKcs share some functions, their roles and, thus, the phenotypes associated with their perturbation differ (*7*, *9*). Unlike ATM and DNA-PKcs, ATR is essential in proliferating cells; therefore, ATR knockout leads to proliferative failure in culture and early embryonic lethality (*12*, *13*).

SLE is a heterogeneous and potentially severe autoimmune disease, wherein the interplay between environmental and genetic factors leads to perturbation of complex biological networks that culminate in immune dysregulation (*14*). B cell aberrancies are critically involved in all levels of SLE pathophysiology, contributing to immune deregulation (*15*–*18*). More specifically, B cell key pathogenic features in SLE include the following: (i) a hyperactive profile with increased antigen presentation capability; (ii) expanded populations of plasmablasts, memory, double-negative memory, and transitional cells; (iii) aberrancies in both pro- and anti-inflammatory cytokine secretion, highlighted by increased interleukin-10 (IL-10), IL-6, IL-4, and tumor necrosis factor–β (TNF-β) and decreased IL-2 production; and (iv) increased secretion of pathogenic autoantibodies (*15*, *18*, *19*). This abnormal B cell function in SLE is affected by type I interferon (IFN) correlating with an IFN molecular signature present especially in patients with moderate to severe lupus (*20*, *21*). Despite the central role of B cells in SLE pathogenesis, the mechanisms driving their deregulation are only partly understood; current therapeutic approaches that target B cells [e.g., rituximab and belimumab (*22*)] eliminate the entire B cell population, leading to immunosuppression and attenuate humoral immune responses to vaccines (*23*).

On the basis of the above, a currently unmet need is the understanding of the molecular events that operate in pathogenic B cell clones, which will allow their selective targeting, leaving the healthy B cell repertoire unaffected to mediate immune protection. Although B cells are prone to DDR errors because DDR is co-opted in antibody diversification (*8*) and accumulating evidence reports inefficient DDR in leukocytes from patients with SLE, the molecular links of B cell DDR with the pathophysiology of autoimmune diseases are ill-defined (*3*–*5*, *24*–*26*). ATM down-regulation in B cells of patients with RA mediates a pro-ostoeclastogenic B cell phenotype driving erosive disease (*6*). To this direction, elucidation of the molecular pathways that underlie DDR in pathogenic B cells may facilitate the design of rational therapies for patients with SLE.

Here, we used proteomic and transcriptomic analyses of B cells from patients with SLE and healthy individuals to demonstrate a dominant DDR in SLE B cells. Experimental screening of the major DDR pathways identified a profound ATR/Chk1 axis activation in SLE B cells as compared to healthy B cells. IFN-α exposure of healthy B cells (SLE-like environment) mimicked the DDR signature of SLE B cells, while ex vivo suppression of ATR activity reversed cells’ pathogenic phenotype. We also provide evidence that IFN regulatory factor 1 (IRF1), an important contributor to type I IFN signature in SLE, is part of the DDR machinery mediating ATR pathway activation in IFN-α–treated B cells. Our data indicate that IFN-α–mediated, IRF1-driven ATR machinery contributes to SLE pathogenesis by promoting B cell hyperactivity and demonstrate a previously unidentified pathway by which type I IFN promotes humoral immune responses.

## RESULTS

### B cells of patients with SLE exhibit enriched DDR

**Fig. 1.**
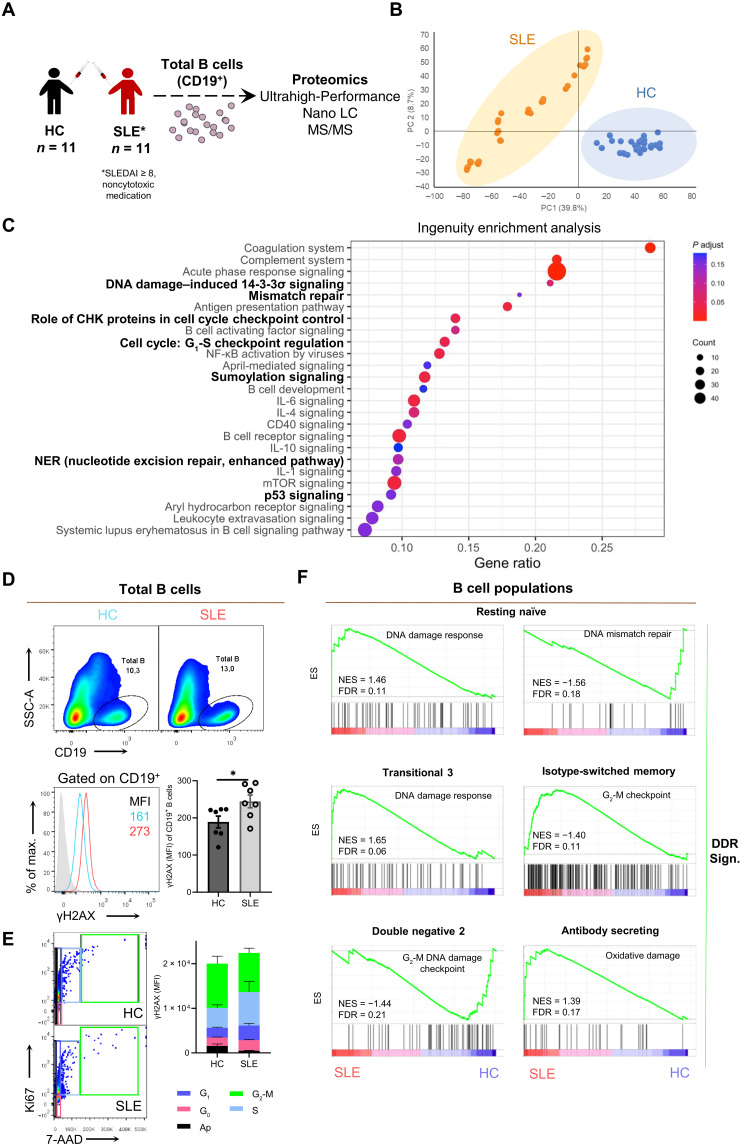
Proteomic and transcriptomic analyses identify DDR as a feature of SLE B cells. (**A**) B cells were isolated using magnetic bead–based approach from the peripheral blood of patients with SLE and HC (*n* = 11 individuals per group) for proteomic analysis. (**B**) Principal components analysis (PCA) using the expression of all proteins for patients with SLE and HC in technical triplicates. (**C**) IPA using the 1094 differentially expressed proteins [false discovery rate (FDR) < 0.05] between SLE and HC. Selected enriched canonical pathways (FDR < 0.2) are shown. DDR-related pathways are indicated in bold. (**D**) Flow cytometric analysis of peripheral blood mononuclear cells (PBMCs) gated on CD19^+^ for assessing DDR activation in B cells of patients with SLE and HC (*n* = 7 individuals per group) with γΗ2ΑΧ. Representative plots of frequencies and histogram showing overlay of unstained cells (gray), stained SLE (red), and HC (blue) cells are depicted. Statistical significance was obtained by unpaired Student’s *t* test, *P < 0.05. (**E**) Flow cytometric analysis for assessing γΗ2ΑΧ expression across cell cycle phases in magnetic bead–isolated B cells of patients with SLE and HC (*n* = 6 individuals per group). Cell cycle analysis was performed using Ki67 and 7-AAD. The comparison involves the same phase of the two groups (SLE and HC). No statistical significance was noted in any cell phase between the two groups [two-way analysis of variance (ANOVA)]. (**F**) Gene set enrichment analysis (GSEA) plots for DDR alterations across B cells subsets (resting naïve, transitional 3, isotype-switched memory, double negative 2, and antibody-secreting) of patients with SLE (*n* = 9) compared to HC (*n* = 12) using a published RNA sequencing (RNA-seq) dataset (*15*). LC, liquid chromatography; MS/MS, tandem mass spectrometry; ES, enrichment score; NES, normalized enrichment score; FDR < 0.25 cutoff. MFI, mean fluorescent intensity. Results are presented as means ± SEM.

To identify previously unknown mechanisms with functional importance related to the B cell pathogenic role in SLE, we first assessed the proteomic profile of total B cells isolated from the peripheral blood of patients with active SLE (SLEDAI ≥8) and age/sex-matched healthy controls (HC) (Fig. 1A and table S1 with demographics). The generated dataset contained a total number of 6449 of master protein groups that were further filtered on the basis of the presence of at least two peptides resulting in a protein list of 4995 proteins. Principal components analysis of total proteins exhibited a strong separation between the two groups (Fig. 1B). Both ingenuity enrichment analysis (IPA) and STRING pathway analysis revealed key known features involved in SLE pathogenesis being significantly deregulated in SLE B cells compared to HC, such as complement activation, circulating antibodies regulation, cytokine production (IL-1, IL-4, IL-6, and IL-10), antigen presentation, inflammatory response, nuclear factor κB (NF-κΒ) activation, and mammalian target of rapamycin (mTOR) signaling (Fig. 1C, fig. S1, and table S2). Notably, B cells from SLE subjects exhibited enriched response to DNA damage stimulus. In particular, DNA repair, p53 signaling, G_1_-S checkpoint regulation, and other DDR-related pathways were overrepresented in the proteomic signature of SLE B cells compared to HC (Fig. 1C and fig. S1). To validate the enriched DDR, we performed flow cytometry assays for γH2AX, a classic indicator of DDR activation (*27*). We detected increased levels of γH2AX in both human and murine (NZB/W-F1) SLE total peripheral B cells as compared to HC, further supporting the exacerbated DDR in SLE B cells (Fig. 1D and fig. S2). Next, we asked whether enriched DDR in SLE is a specific feature of B cells or a general feature of immune cell populations. Thus, the expression levels of γH2AX were also evaluated in peripheral T helper (CD4^+^), T cytotoxic (CD8^+^), T regulatory (CD4^+^CD25^+^Foxp3^+^), classical monocytes (HLA-DR^+^CD14^+^CD16^−^), intermediate monocytes (HLA-DR^+^CD14^+^CD16^+^), nonclassical monocytes (HLA-DR^+^CD14^−^CD16^+^), and neutrophils (CD66b^+^) of individuals with SLE and HC. Among the diverse SLE-derived immune cells examined, B cells demonstrated the most prominent increase of γΗ2ΑΧ expression compared to HC, as revealed by both flow cytometry and confocal microscopy assays (figs. S3 and S4).

Because DDR comprises cell cycle checkpoint control, we sought to examine whether elevated γΗ2ΑΧ expression in SLE B cells is associated with a specific phase of the cell cycle. Thus, we performed cell cycle analysis via flow cytometry upon simultaneous assessment of Ki67 proliferation–specific marker, 7-AAD cellular DNA content marker, and γΗ2ΑΧ in freshly isolated B cells from patients with SLE and HC. Despite the fact that γΗ2ΑΧ was significantly overexpressed in the SLE compared to HC B cells, no statistically significant difference was noted in any cell phase, suggesting that the SLE-associated up-regulation of γΗ2ΑΧ may be independent of cell cycle phase (Fig. 1E and fig. S5, A and B).

Last, to investigate whether the proteomic data of overall deregulated DDR could be extrapolated to the transcriptome and if a specific B cell subpopulation prevails in the DDR signature, we performed gene set enrichment analysis (GSEA) of B cell subsets from patients with SLE and HC, exploiting a publicly available RNA sequencing (RNA-seq) dataset (*15*). The results indicated that almost all major SLE B cell subpopulations are involved in the altered DDR, including resting naïve, transitional 3, isotype-switched memory, double negative 2, and antibody-secreting B cells (Fig. 1F). Together, these data indicate that B cells exhibit a profound DDR in individuals with SLE.

### Activation of ATR pathway drives DDR in SLE B cells

**Fig. 2.**
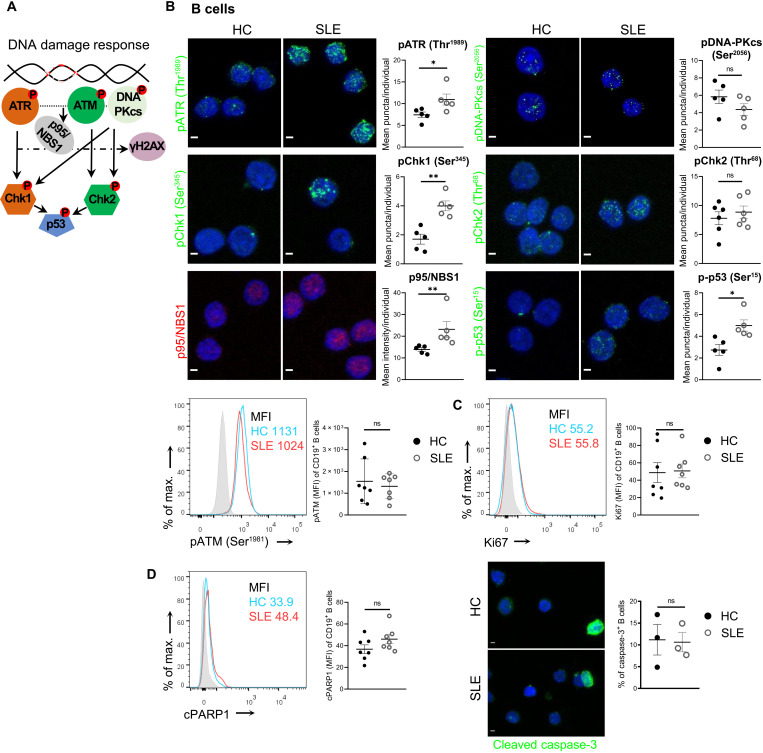
ATR/Chk1 DDR pathway is activated in SLE B cells. (**A**) Schematic representation of the main DDR pathways being the ATR/Chk1, ATM/Chk2, and DNA-PKcs. (**B**) Investigation of DDR contributors and immunofluorescence confocal microscopy images or representative histogram of stained SLE and HC B cells [CD19^+^, isolated by fluorescence-activated cell sorting (FACS) or magnetic beads]. Representative confocal images for pATR (Thr^1989^; green; *n* = 5 individuals per group), pChk1 (Ser^345^; green; *n* = 5 individuals per group), p95/NBS1 (red; *n* = 5 individuals per group), pATM (Ser^1981^; *n* = 7 individuals per group), pDNA-PKcs (Ser^2056^; green; *n* = 5 individuals per group), pChk2 (Thr^68^; green; *n* = 6 individuals per group), and p-p53 (Ser^15^; green; *n* = 5 individuals per group). Analyzed results for pATR, pChk1, pDNA-PKcs, pChk2, and p-p53 are depicted as mean puncta/cell per individual, for pATM as MFI per individual, and for p95/NBS1 as mean intensity/cell per individual. For pATM, representative histogram showing overlay of unstained cells (gray), stained SLE (red), and stained HC cells (blue) is depicted. Scale bars, 2 μm. (**C**) Assessment of proliferation (Ki67) and (**D**) apoptosis (cleaved PARP1 and cleaved caspase-3) in SLE and HC magnetic bead–isolated B cells via flow cytometry (*n* = 7 individuals per group) and/or confocal microscopy. Representative histograms showing overlay of unstained cells (gray), stained SLE cells (red), and stained HC cells (blue) are depicted. For cleaved caspase-3 staining, the positive signal is depicted with green, and cells are quantified as positive or negative using 4′,6-diamidino-2-phenylindole (DAPI) nuclear staining (blue; *n* = 3 individuals per group). Scale bar, 2 μm. For all cases, results are expressed as means ± SEM. Statistical significance was obtained by unpaired Student’s *t* test, *P* ≥ 0.05 [not significant (ns)], *P < 0.05, and **P < 0.01.

Next, we sought to characterize the specific molecular mechanism underlying the DDR by examining master kinase proteins and components of DDR, namely, ATR, ATM, DNA-PKcs, Chk1, Chk2, p53, and p95/NBS1 (*7*), in SLE and HC B cells (Fig. 2A). Because the activation and transduction of the DDR signaling usually require protein phosphorylation, most targets were investigated in their phosphorylated form (*28*). We found increased levels of pATR (Thr^1989^), pChk1 (Ser^345^), p-p53 (Ser^15^), and p95/NBS1 in SLE compared to HC B cells, but no differences in pATM (Ser^1981^), pChk2 (Thr^68^), and pDNA-PKcs (Ser^2056^) levels via immunofluorescence microscopy or flow cytometry (Fig. 2B). The activation of the ATR pathway in SLE B cells was also confirmed by Western blot analysis in separate individuals (fig. S6). These data indicate that the ATR/Chk1 pathway is mainly activated in SLE B cells, while ATM/Chk2 and DNA-PKcs are not critically involved. Moreover, to provide additional evidence that ATR-mediated DDR pathway is specific to SLE and not secondary to the inflammatory milieu of any autoimmune disease, we examined pATR protein levels in B cells isolated from the periphery of patients with ankylosing spondylitis (AS) (table S1 with demographics). AS is an autoinflammatory disease, wherein, unlike SLE, B cells do not have a major role in pathogenesis and type Ι IFN signature is absent (*29*). Nonsignificant changes were noted in pATR protein levels in AS-derived B cells compared to HC (fig. S7).

DDR machinery may induce apoptosis and/or proliferation, and in particular, ATR/Chk1 activation is anticipated to affect cell proliferation (*30*, *31*). However, neither proliferation (Ki67) nor apoptosis [cleaved poly(ADP-ribose) polymerase 1 (PARP1) and cleaved caspase-3] differed in SLE B cells when compared to HC, indicating that ATR/Chk1 pathway deregulation may not have a significant impact on these processes in SLE and that other cell responses may be affected (Fig. 2, C and D). Although proliferation rate did not differ significantly between SLE and HC B cells (Fig. 2C), the statistically significant decrease of G_1_ phase–derived B cells (fig. S5B), along with the proteomic and transcriptomic data indicating deregulated G_1_-S and G_2_-M checkpoints (Fig. 1C and fig. S1) in SLE compared to HC, prompted us to further investigate ATR activity in proliferative (S phase) and mitotic (G_2_-M phase) B cells. To this end, we examined pATR expression levels in S (EdU^+^) and G_2_-M (pH3^+^) phase–derived cells, following ex vivo activation (IL-21/CpGb/sCD40L cocktail for survival and mild induction of proliferation) (*32*–*38*) and EdU exposure of peripheral total B cells from patients with SLE and HC (fig. S8A). The results indicated that at both SLE and HC, the ATR pathway was up-regulated in EdU^+^ compared to EdU^−^ B cells, while pH3^+^ cells exhibited increased pATR expression compared to pH3^−^ cells only in lupus environment. Nonetheless, the pATR expression in EdU^+^ SLE B cells was significantly higher compared to EdU^+^ HC B cells, while it did not present a significant change in pH3^+^ SLE B cells when compared to pH3^−^ SLE B cells. These data suggest that although, eventually, overall proliferation rate is not altered, ATR may have a more active role in the growth and division of B cells in SLE compared to HC (fig. S8, B and C).

### Inhibition of ATR alters cytokine production of IFN-α–treated B cells

**Fig. 3.**
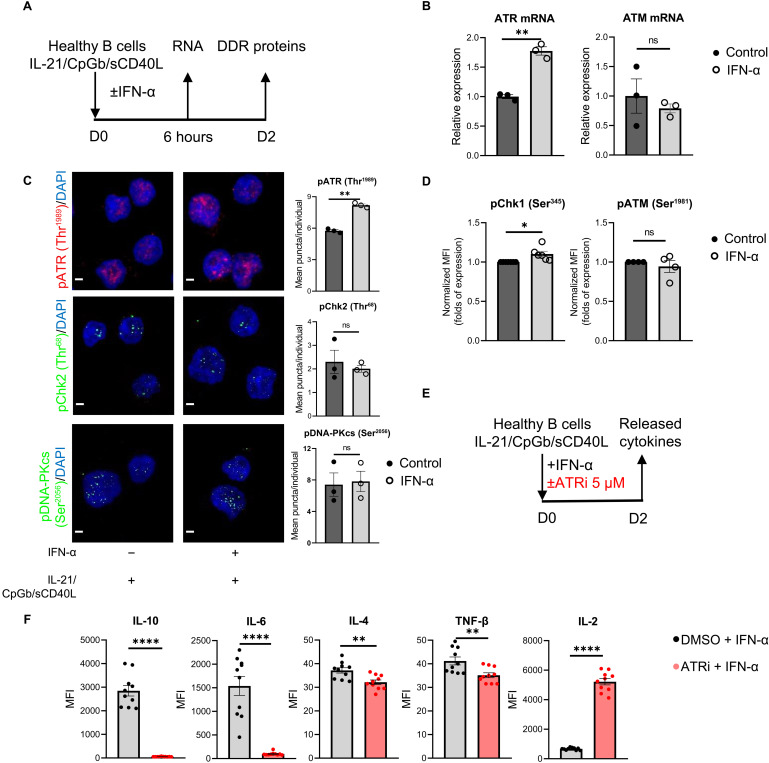
ATRi alters release of cytokines in IFN-α–treated B cells. B cells were isolated from the peripheral blood of healthy individuals using magnetic bead–based approach and cultured with IL-21/CpGb/sCD40L survival and mild proliferation stimuli cocktail in the presence or absence (control) of IFN-α (850 U/ml) to mimic lupus environment ex vivo and with ATRi (5 μΜ) or dimethyl sulfoxide (DMSO), depending on the experiment. (**A**) Schematic representation of IFN-α–treated B cells ex vivo experiments. (**B**) Relative expression levels of ATR and ATM mRNA in control and IFN-α–treated conditions measured with quantitative real time reverse transcription polymerase chain reaction (RT-PCR; *n* = 3 individuals per condition; paired Student’s *t* test). (**C**) Control and IFN-α–treated B cells were stained and quantified (mean puncta/cell per individual) for pATR (Thr^1989^; red), pChk2 (Thr^68^; green), pDNA-PKcs (Ser^2056^; green) and labeled with DAPI nuclear staining (blue; *n* = 3 individuals per condition; paired Student’s *t* test). Scale bars, 2 μm. (**D**) Assessment of pChk1 (Ser^345^) and pATM (Ser^1981^) in control and IFN-α–treated B cells via flow cytometry followed by normalization of MFI to the MFI of control cells (unpaired Student’s *t* test). Cells were gated on CD19^+^. (**E**) Schematic representation of ATRi (berzosertib) experiment at IFN-α–treated B cell ex vivo to assess cytokine production. Cell culture supernatants were collected following 2 days (D2) of culture. (**F**) Detection of released cytokines (IL-10, IL-6, IL-4, TNF-β, and IL-2) using LEGENDplex technology through flow cytometry at D2 of culture (*n* = 10 individuals per condition; paired Student’s *t* test). Results are expressed as means ± SEM. *P* ≥ 0.05 (ns), *P < 0.05, **P < 0.01, and ****P < 0.0001.

To clarify the functional involvement of ATR-mediated DDR mechanism in B cells in an SLE-like environment, total B cells were isolated from the periphery of healthy individuals and exposed to IFN-α, the main type I IFN, following their ex vivo activation (Fig. 3A). ATR mRNA, pATR, and pChk1 protein levels were up-regulated upon IFN-α administration, whereas no significant alterations in ATM mRNA, pATM, pChk2, and pDNA-PKcs expression were noted (Fig. 3, B to D). Moreover, IFN-α–treated B cells exhibited ATR pathway activation when being in S and G_2_-M phases of the cell cycle similar to SLE B cells (fig. S8, A to C). Therefore, IFN-α exposure of B cells recapitulated the cardinal feature of up-regulated ATR-mediated DDR pathway noted in SLE being a sufficient experimental setup to mimic the SLE environment throughout this study.

To delineate the role of ATR in SLE pathogenesis, we first sought to profile cytokine production following ATR inhibition in IFN-α–treated B cells, considering that B cells represent a rich source of various cytokines, which are broadly perturbed in SLE. To date, whether ATR-mediated DDR pathway is involved in the production and secretion of cytokines remains largely unknown. To this end, berzosertib (ATRi), a pharmaceutical inhibitor that restrains ATR function by blocking Chk1 activation (*39*), was added to IFN-α–treated healthy B cells ex vivo (Fig. 3E and fig. S9). We then assessed the release of important cytokines [TNF-α, IL-13, IL-4, IL-10, IL-6, IL-2, TNF-β, IFN-γ, IL-17A, IL-12p70, a proliferation-including ligand (APRIL), B cell–activating factor (BAFF), and CD40 ligand (CD40L)] for both SLE pathogenesis and B cell growth in IFN-α–treated B cells with or without ATRi. IL-10, IL-6, IL-4, and TNF-β levels were significantly decreased upon ATRi (Fig. 3F), while the levels of IL-2, IL-12p70, CD40L, BAFF, APRIL, and IFN-γ were increased (Fig. 3F, fig. S10). Together, these data demonstrate that ATRi may reprogram the cytokine profile of SLE-like B cells.

### Inhibition of ATR reduces the immunogenicity of IFN-α–treated B cells

**Fig. 4.**
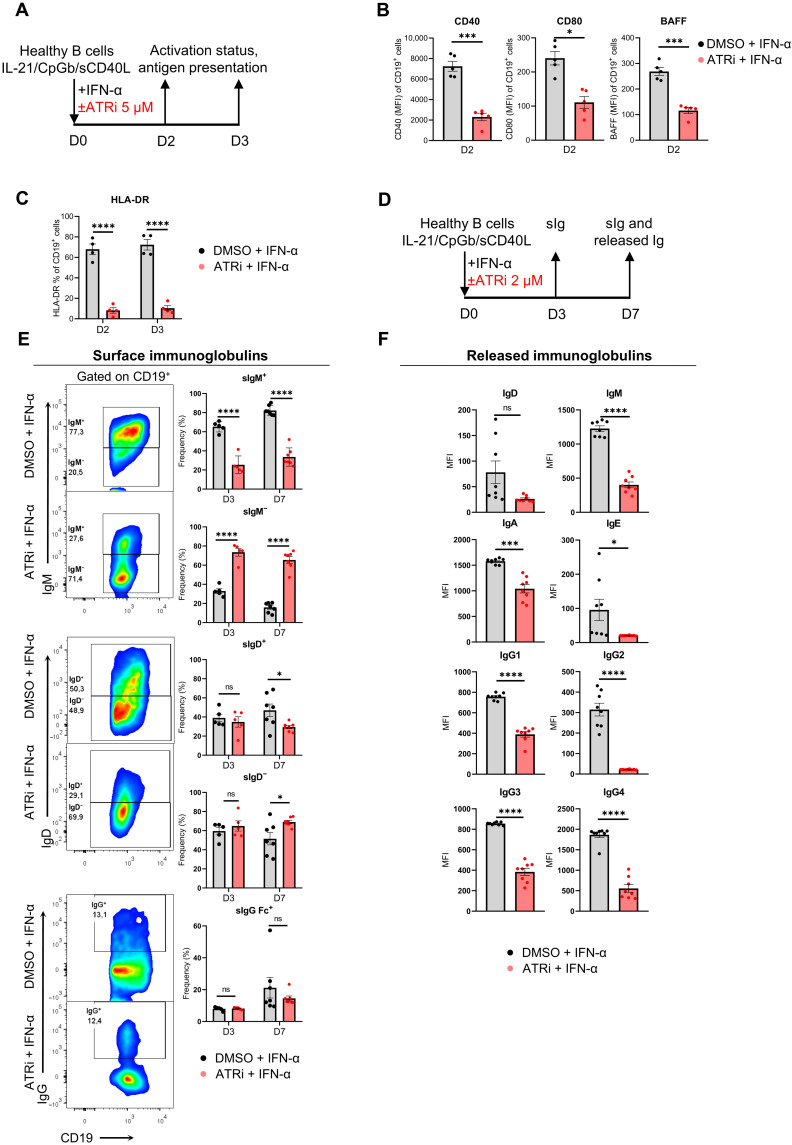
ATRi restrains activation and antibody formation in IFN-α–treated B cells. B cells were isolated from the peripheral blood of healthy individuals using magnetic bead–based approach and cultured with IL-21/CpGb/sCD40L survival and mild proliferation cocktail in the presence of IFN-α (850 U/ml) and in the presence or absence of ATRi or DMSO (control) for 2 (D2), 3 (D3), and 7 (D7) days depending the experiment. (**A**) Schematic representation of ATRi experiment at IFN-α–treated B cells ex vivo for assessing cell activation status (ATRi, 5 μΜ). Quantification of flow cytometry retrieved data gated on CD19^+^ of (**B**) CD40 (*n* = 5 individuals per condition), CD80 (*n* = 5 individuals per condition), BAFF (*n* = 5 individuals per condition), and (**C**) HLA-DR (*n* = 4 individuals per condition). Paired Student’s *t* test was applied in all cases. (**D**) Schematic representation of ATRi experiment (ATRi, 2 μΜ) at IFN-α–treated B cell ex vivo to assess surface and released immunoglobulins. (**E**) Representative flow cytometry gating for surface IgM, IgD, and IgG at D7 and quantification at D3 and D7 (*n* = 5 to 7 individuals per condition; two-way ANOVA). (**F**) Detection of released immunoglobulins (IgM, IgD, IgA, IgE, IgG1, IgG2, IgG3, and IgG4) using LEGENDplex technology in collected cell culture supernatants through flow cytometry (*n* = 8 individuals per condition; paired Student’s *t* test). Results are expressed as means ± SEM. *P* ≥ 0.05 (ns), *P < 0.05, ***P < 0.001, and ****P < 0.0001.

Results thus far imply that ATR-mediated DDR pathway is critically involved in the production of cytokines by B cells in SLE, plausibly driving SLE pathophysiology in an autocrine fashion. Moreover, SLE B cells are known to adopt an activated status and enhanced antigen presentation ability followed by increased plasmablast formation and antibody production (*15*, *18*, *40*–*42*). To this end, we evaluated cell activation (CD40, CD80, and surface BAFF) and antigen presentation capability [human leukocyte antigen–DR (HLA-DR)] across different time points during inhibition of ATR activity in IFN-α–treated B cells (Fig. 4, A to C, and fig. S11, A and B). Upon inhibition of ATR, activation and antigen presentation by HLA-DR were down-regulated in IFN-α–treated B cells, even when ATRi was administered at low dose (fig. S12). Notably, this phenotype may be ATR dependent and Chk1 independent because pharmaceutical inhibition downstream of ATR-Chk1 interactive regulation with CHIR-124 (Chk1i, a potent inhibitor of Chk1 activity) did not affect these cell properties (figs. S12C and S13). Therefore, ATR activation per se plays a critical role in the activation of IFN-α–treated B cells, and the aforementioned variable cytokine expression upon ATR inhibition does not reflect a hyperactive B cell population.

**Fig. 5.**
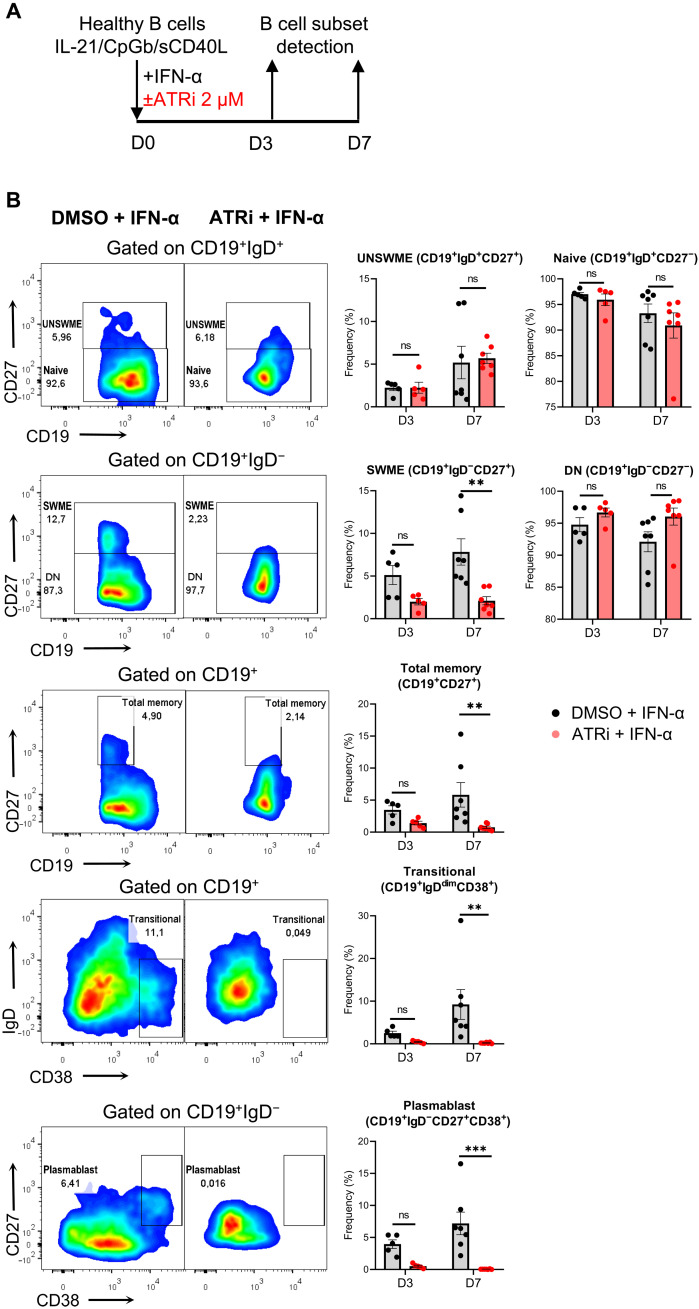
ATRi impedes plasmablast formation of IFN-α–treated B cells. B cells were isolated from the peripheral blood of healthy individuals (*n* = 5 to 7) using magnetic bead–based approach and cultured with IL-21/CpGb/sCD40L survival and mild proliferation cocktail in the presence of IFN-α (850 U/ml) and in the presence or absence of ATRi or DMSO (control) for 3 (D3) and 7 (D7) days. (**A**) Schematic representation of ATRi experiment (ATRi, 2 μΜ) at IFN-α–treated B cells ex vivo for assessing B cell subsets differentiation status. (**B**) Representative flow cytometry gated cell populations at D7 and quantification of corresponding populations at D3 and D7 (D3, *n* = 5 individuals per condition; D7, *n* = 7 individuals per condition). The B cell populations were immunophenotyped in flow cytometry as follows: unswitched memory (UNSWME) as CD19^+^IgD^+^CD27^+^, naïve as CD19^+^IgD^+^CD27^−^, switched memory (SWME) as CD19^+^IgD^−^CD27^+^, double negative (DN) as CD19^+^IgD^−^CD27^−^, total memory as CD19^+^CD27^+^ transitional as CD19^+^IgD^dim^CD38^+^, and plasmablasts as CD19^+^IgD^−^CD27^+^CD38^+^. Results are expressed as means ± SEM. *P* ≥ 0.05 (ns), **P < 0.01, and ***P < 0.001 (two-way ANOVA).

Next, we investigated whether antibody (i.e., immunoglobulin) production is affected because their production may follow the release of cytokines, as in the case of CD40L, BAFF and APRIL but, on the other hand, may also be attenuated due to decrease of proinflammatory cytokines, such as IL-6 and TNF-β. The results showed that surface immunoglobulin M (sIgM) was decreased by day 3 of culture; sIgD was decreased by day 7, whereas sIgG did not exhibit any significant difference in IFN-α–treated B cells exposed to ATRi compared to the unexposed (Fig. 4, D and E). Furthermore, released IgM, IgA, IgE, IgG1, IgG2, IgG3, and IgG4 were strongly decreased in IFN-α–treated B cells with ATRi (Fig. 4, D and F), suggesting that ATR DDR pathway has a central role in antibody formation. Last, because of the decreased levels of both surface IgM and IgD and released immunoglobulins by IFN-α–treated B cells with ATRi, we anticipated a reduction in isotype-switched and antibody-secreting B cells. Therefore, we sought to investigate B cell subset formation (Fig. 5A). At day 7 of culture, IFN-α–treated B cells with ATRi exhibited markedly decreased levels of isotype-switched memory (SWME) cells (CD19^+^IgD^−^CD27^+^), total memory cells (CD19^+^CD27^+^), transitional cells (CD19^+^IgD^dim^CD38^+^), and plasmablasts (CD19^+^IgD^−^CD27^+^CD38^+^), while isotype-unswitched memory (UNSWME) (CD19^+^IgD^+^CD27^+^), naïve (CD19^+^IgD^+^CD27^−^), and double-negative cells (CD19^+^IgD^−^CD27^−^) remained almost unaffected compared to those not treated with ATRi (Fig. 5, A and B).

Last, we examined whether ATRi influences SLE B cell responses in additional ways such as by affecting the cell cycle profile. Our data in IFN-α–treated B cells showed that upon ATRi, there were significantly increased G_0_-derived B cells compared to dimethyl sulfoxide (DMSO) condition (control), an observation consistent with the reduced cell cluster formation as observed under the upright microscope (fig. S14). Therefore, by introducing the SLE-like B cells to a resting state (G_0_), their immune responses declined as expected.

Collectively, these results suggest that inhibition of ATR activity reduces the immunogenicity of IFN-α–treated B cells by (i) decreasing the release of IL-10, IL-6, IL-4, and TNF-β levels while increasing the release of IL-12p70 and IL-2; (ii) attenuating cell activation; (iii) restraining immunoglobulin formation; (iv) inhibiting class switching and formation of plasmablasts; and (v) arresting them in resting state.

### IRF1 directly interacts with the promoter sequence of *ATR* gene in IFN-α–treated B cells and modulates ATR activity

**Fig. 6.**
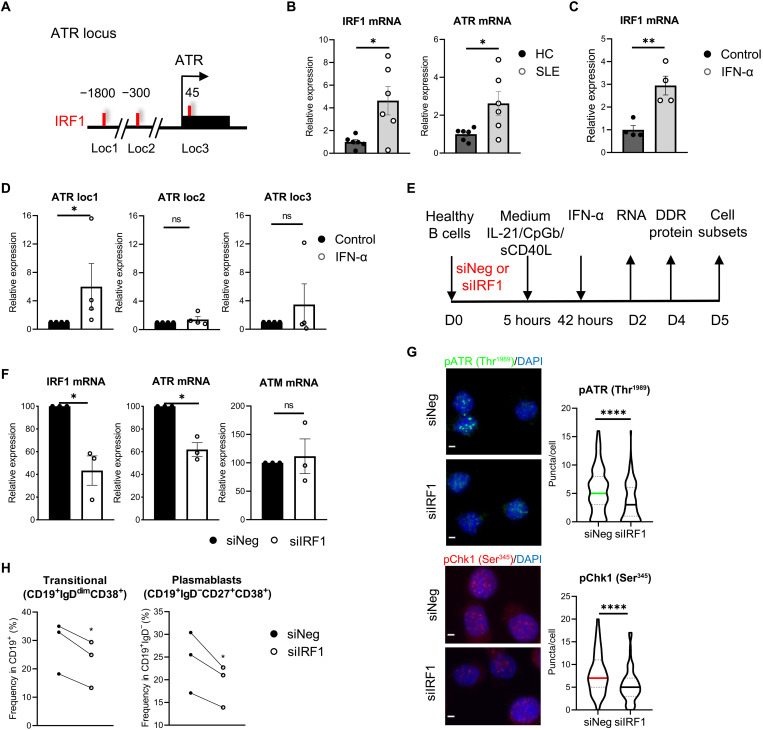
IRF1 directly interacts with the promoter sequence of *ATR* gene in IFN-α–treated B cells and modulates ATR activity. (**A**) Schematic representation of the *ATR* gene locus around the transcription start site (TSS, arrow; coding region, black box). Numbers above the schematic drawing denote the distance from the TSS and the predicted binding sites of IRF1 (EPD database). (**B**) IRF1 and ATR mRNA levels in magnetic bead–isolated B cells of patients with SLE and HC (*n* = 6 per group) analyzed by quantitative real-time RT-PCR (unpaired Student’s *t* test). (**C**) IRF1 mRNA levels as measured with quantitative real time RT-PCR (*n* = 4 individuals per condition) in IFN-α–treated B cells (paired Student’s *t* test). Cells were cultured with IL-21/CpGb/sCD40L survival and mild proliferation cocktail and IFN-α (850 U/ml) for 2 days. (**D**) ChIP analysis of the binding sites of IRF1 to *ATR* gene locus. ChIP experiments were performed using anti-IRF1 antibody (a-IRF1) or a control antibody (IgG) in chromatin isolated from IFN-α–treated B cells (*n* = 4 healthy individuals; unpaired Student’s *t* test). (**E**) Schematic representation of silencing IRF1 expression in IFN-α–treated B cells ex vivo using siIRF1 or siNeg (control). (**F**) Relative expression levels of IRF1, ATR, and ATM mRNA of siNeg and siIRF1 conditions measured with quantitative real time RT-PCR (*n* = 3 individuals per condition). (**G**) SiNeg and siIRF1 B cells were stained and quantified (puncta/cell) for pATR (Thr^1989^; green) or pChk1 (Ser^345^; red) and labeled with DAPI (blue; *n* = 3 individuals per condition; unpaired Student’s *t* test). Scale bars, 2 μm. (**H**) Flow cytometric analysis for transitional (CD19^+^IgD^dim^CD38^+^) and plasmablast (CD19^+^IgD^−^CD27^+^CD38^+^) B cells upon siNeg and siIRF1 (*n* = 3 individuals per condition; paired Student’s *t* test). For all cases, *P* ≥ 0.05 (ns), *P < 0.05, **P < 0.01, and ****P < 0.0001. For (B) to (D) and (F), results are expressed as means ± SEM.

To investigate the molecular mechanism by which SLE-like B cells undergo direct regulation of ATR by IFN-α, we performed chromatin immunoprecipitation (ChIP) experiments for binding sites of IRF1 on *ATR* regulatory regions, as predicted by in silico analysis [Eucaryotic Promoter Database (EPD) database] (Fig. 6A). Both ATR and IRF1 mRNA levels were increased in B cells of patients with SLE (Fig. 6B), as well as in healthy B cells upon IFN-α administration (Figs. 3B and 6C). Following ChIP reactions with anti-IRF1 antibody or control IgG in IFN-α–treated B cells, we identified an IRF1-binding event (i.e., *loc1*) on *ATR* gene promoter close to the transcription start site of *ATR* gene that was specifically enriched for anti-IRF1 reactions (Fig. 6D). These data suggest that IRF1 may directly interact with *ATR* regulatory sequences to transcriptionally mediate its expression.

To directly assess whether IRF1 binding on *ATR* gene locus has a regulatory role, we performed knockdown of IRF1 with small interfering IRF1 RNA (siIRF1) or negative control (siNeg) in IFN-α–treated B cells and examined ATR mRNA, ATR, pATR (Thr^1989^), and pChk1 (Ser^345^) protein levels (Fig. 6, E to G, and fig. S15). ATM mRNA levels were also checked as a control to ensure IRF1 specificity for ATR (Fig. 6F). Knockdown of IRF1 in these cells down-regulated ATR mRNA, ATR, pATR, and pChk1 protein levels whereas retained ATM mRNA levels (Fig. 6, F and G, and fig. S15). IRF1 silencing suppressed the development of both transitional and plasmablast B cell subsets, similar to the results obtained following pharmaceutical ATR inhibition (Fig. 6H and fig. S16). Overall, these results support that IRF1 mediates ATR activity, explaining the DDR observed in IFN-α–treated and SLE B cells.

## DISCUSSION

Although DDR has been implicated in all facets of inflammatory states, little is known about how its deregulation may render a specific immune cell population pathogenic. The DDR-driven molecular mechanisms in autoimmunity remain poorly understood. B cells have a pivotal role in the development of SLE disease, yet the driving factors for their pathogenicity are elusive. In this study, using proteomic and transcriptomic analyses of B cells from patients with SLE, we reveal a deregulated DDR. More specifically, we describe a previously unidentified mechanism associated with B cell dysfunction, which entails the engagement of ATR-mediated pathway being specifically enriched in SLE B cells. Our ex vivo data revealed that in the course of an autoimmune response driven by type I IFN, ATR/Chk1 pathway was triggered and that targeted pharmaceutical inhibition of ATR activity restrained key features of SLE pathophysiology including B cell activation, plasmablast formation, antibody production, and proinflammatory cytokines release. Last, we provide evidence that this ATR overactivation is mediated by direct molecular interaction with IRF1. Together, these data link the characteristic type I IFN signature of SLE with the up-regulation of ATR/Chk1 pathway and the induction of pathogenic B cells mediated via IRF1. Pharmacologic targeting of ATR alleviates the pathogenic cell features pointing to the ATR pathway as a potential therapeutic target in SLE.

In accordance with the literature, up-regulated DDR was also observed in SLE T cytotoxic and neutrophils (*43*, *44*), but B cells exhibited the most prominent DDR increase. Our data derive from both naïve and active-phase patients, suggesting that these findings are likely mirroring a primary pathogenetic mechanism in SLE. Although roles of DDR components have been previously proposed in pathogenic cells in processes associated with SLE, including their implications in V(D)J recombination, somatic hypermutation, and cell death (*3*, *5*, *26*), a comprehensive understanding of their specific contribution in autoreactive B cell responses is ill-defined. Here, we expand this knowledge by identifying the ATR-mediated DDR pathway deregulation in B cells and link this pathway to their pathogenic potential. While our data highlight a clear increase in ATR/Chk1 pathway activation, with no alterations in ATM/Chk2 and DNA-PKcs in SLE B cells, a study by Taher *et al.* (*45*) reported a reduction in ATR activation and an increase in ATM activation in B cells from patients with SLE. In contrast to this study, our experimental design consisted of patients who had both high disease activity and were not under cytotoxic medication to avoid the possibility that the observed DDR may be affected by these drugs. In agreement with our findings, studies on B cell lymphomagenesis, in the context of Epstein-Barr virus (EBV) infection, demonstrate the involvement of ATR/Chk1 in abnormal B cell responses (*46*–*50*). In particular, in a recent study examining DDR activation in EBV-exposed B cells, ATR/Chk1, but not ATM/Chk2 pathway, was activated, while specific targeting of ATR was able to suppress B cell aberrant function upon the infection (*50*). Patients with SLE have increased risk of B cell lymphoma, and EBV virus has been acknowledged as a potential inducer of lupus autoimmunity, indicating that these conditions share common pathogenetic mechanisms (*51*). It is tempting to speculate that ATR/Chk1 deregulation could drive the abnormal B cell profile in these conditions. On the other hand, ATM/Chk2 pathway is of interest in B cell autoimmunity because it has been recently reported to drive B cell pathogenicity in a group of patients with RA exhibiting severe disease (*6*). ATM deregulation both depends and triggers IFN signaling following DDR perturbations and infections (*52*, *53*). These data coupled with our findings suggest that distinct DDR pathways may drive differential pathogenic responses of the same cell population depending on the type of autoimmune environment. Our study adds a new layer for ATR-mediated pathway in autoimmunity and, specifically, in regulating B cell responses in SLE.

Targeting B cells or B cell–expressing molecules has generated promising results in patients with SLE (*54*). The most recently approved therapies for SLE include the monoclonal antibody against BAFF and targeting type I IFN through a receptor monoclonal antibody that blocks the action of type I IFNs (IFN-α and IFN-β) (*55*). These and other standard therapies for SLE (such as anti-CD20 drugs and cyclophosphamide) deplete most B cells including those involved in antiviral immunity, generating a major limitation in the face of the current corona virus disease 2019 pandemic (*56*). Patients with SLE have a currently unmet medical need for more effective and safer therapies, suggesting that there is a missing link between the disease pathogenesis and the available therapies. We envisage that this link could be the aberrant DDR of B cells that is not affected by the current drugs. Targeting of ATR-mediated DDR pathway in SLE B cells could halt the pathogenic B cell responses while potentially sparing the protective responses.

Our data demonstrate that blockade of ATR activity in IFN-α–treated B cells was followed by a B cell profile that does not predispose to SLE, such as the reduction in antibody formation; of plasmablasts; and of soluble IL-10, IL-6, IL-4, and TNF-β and the increase of soluble IL-2, yet the released levels of BAFF were increased, while membrane-bound BAFF decreased. Current anti-BAFF therapy, used in SLE, targets both the soluble form and the membrane-bound form of BAFF, with a higher potency for the soluble form (*57*, *58*). While both forms of BAFF are biologically active, whether specific targeting of membrane-bound versus soluble BAFF would yield different clinical outcomes is not established. These findings highlight ATR as a potential therapeutic target for SLE and reveal an integral role of ATR DDR pathway in cytokine production by B cells. It is not clear whether this is a direct effect between ATR activity and signaling of cytokine production or it is due to changes in B cell differentiation status. Rodier *et al.* (*59*) demonstrated that ATM activity is essential for IL-6 production in fibroblast; however, when ATR was inhibited, the production of IL-6 was diminished. In addition, in a recent study (*6*), increased IL-6 secretion was associated with defects in ATM activation by human B cells in RA. Therefore, we conclude that in SLE B cells, cytokine release may be affected by aberrant ATR-mediated DDR and that the specific DDR signaling that modulates cytokine secretion varies depending on the cell type and the immune microenvironment.

Although our findings indicate that both ATR and Chk1 are excessively phosphorylated in SLE B cells compared to HC, the activation and antigen presentation status of SLE-like B cells are affected only when ATR, but not Chk1, is inhibited. It is expected that ATR or Chk1 down-regulation should cause similar alterations because Chk1 activation requires its phosphorylation by ATR. However, it is possible that these two components may not always function as a linear pathway, as supported by several studies presenting differences between the effects of ATRi and Chk1i (*60*–*63*). In this direction, there is evidence that ATR drives class-switch recombination while Chk1 is more involved in normal B cell growth (*64*–*66*), suggesting their distinct contribution in B cell physiology. Speroni *et al.* (*67*) showed that Chk1, but not ATR, drives the progression of replication following ultraviolet irradiation of U2OS cells. An ATR-independent role of Chk1 mediated through its specific interaction with proliferating cell nuclear antigen has been also previously described (*68*, *69*), while Luciani *et al.* (*62*) have reported an ATR-dependent, Chk1-independent, intra–S-phase checkpoint that suppresses initiation of replication. Moreover, in a pioneering study (*63*), the authors have unveiled an ATR role to interact with replication protein A (RPA), a key sensor eliciting DDR following cellular exposure to genotoxic stresses (*70*), independently of Chk1-mediated replication stress responses. Last, the possibility of a different time point–dependent activation between ATR and Chk1 has been investigated; however, it was not confirmed, suggesting that these two components have distinct roles (*60*). Our results uncover a new mechanism of type I IFN contribution to SLE pathogenesis through induction of ATR-mediated DDR by IRF1 in B cells. In support of this, the findings (i) on B cells from patients with AS disease without an IFN signature exhibiting normal levels of ATR activation and (ii) on IFN-α–treated B cells having increased levels of ATR activation suggest that type I IFN is necessary for enriched ATR activity. IRF1 has been noted among the potential drivers of common B cell lymphoid neoplasms and interacts with multiple myeloma oncogene-1 protein (MUM1), which has a role in the progression of B cell lymphoma (*71*–*74*). According to EPD database, other molecules than IRF1 may also directly interact with ATR regulatory regions, suggesting that further investigation may reveal additional mechanisms driving ATR pathway in autoreactive B cells in B cell–mediated diseases.

In our study, IFN-α–treated B cells fail to differentiate sufficiently toward plasmablast upon ATR or IRF1 inhibition, suggesting that the IRF1-ATR axis is critical for the plasmablast formation. In SLE, plasmablast population is often markedly expanded and correlates with disease activity and flares (*18*). Depletion of plasmablast/plasma cells is currently used in SLE therapeutic interventions (*54*). Whereas an involvement of IRF1 has been recently reported in B cells that have differentiated toward plasma cells following lipopolysaccharide stimulation (*75*), a specialized role of ATR in B cell lineage has not been described. Together, these findings suggest that the IRF1-ATR axis is important for B cell differentiation and has a role in the aberrant plasmablast formation in SLE.

In conclusion, we report a specific DDR pathway being overactivated in B cells of patients with SLE, one of the most overreactive cell populations in SLE. We show that ATR-mediated DDR signaling is abnormally regulated in B cells by IRF1, driving pathogenic cell responses. While relevant to understanding SLE pathogenesis and B cell–mediated autoimmune diseases, this may also provide novel insights into the specific regulatory role of ATR signaling in autoimmunity and into its coordination by IFN signaling. Overall our findings propose that targeted manipulation of IRF1-ATR axis in SLE-like B cells may be of therapeutic benefit in SLE. To this end, McNally *et al.* (*1*) have proposed the use of chemical DDR inhibitors to suppress immune responses in antigen-activated T cells in the human autoimmune diseases of hemophagocytic lymphohistiocytosis and MS. In this direction, various selective ATR inhibitors have been tested in phase 1/2 clinical trials for treating solid tumors (*39*, *76*). VE-822, marketed as berzosertib, has shown acceptable safety profile and early efficacy, currently being under evaluation in 18 ongoing clinical trials (e.g., NCT04266912, NCT03641313, and NCT04052555).

### Limitations of study

Targeting of ATR-mediated DDR pathway on pathogenic B cells may ameliorate autoimmune responses but, at the same time, compromise the immune responses to pathogens, although this may be overcome at least in part with vaccination using approved vaccines. Moreover, therapeutic implementation of our findings may be hampered by the lack of protocols to specifically target the ATR pathway on autoreactive B cells as opposed to broad B cell–directed inhibition. Furthermore, generating an appropriate ex vivo experimental pipeline with patient-derived autoreactive B cells, retaining their disease signature, where ATRi effects may be investigated, is an important matter of future studies.

## MATERIALS AND METHODS

### Human subjects

Peripheral blood samples were obtained from patients with SLE [*n* = 51, classified by the 1997 American College of Rheumatology criteria (*77*)] and healthy individuals (*n* = 103). At the time of sampling, all patients had moderate to high disease activity (SLEDAI ≥8,) and, in the vast majority, had not received cytotoxic drugs 12 months before donation. All patients and healthy individuals were recruited through the Rheumatology and Clinical Immunology Unit, fourth Department of Internal Medicine, “Attikon” University Hospital and the Department of Rheumatology, “Asklepieion” General Hospital (both in Athens, Greece). Informed consent was obtained from all individuals before sample collection (Athens, Greece, protocol 10/22-6-2017). All patients omitted any treatment dose for at least 24 hours before blood drawing. To exclude a nonspecific effect of the inflammatory milieu, we used peripheral blood samples from patients with AS (*n* = 4) as additional controls because they present evidence of broad inflammatory response, but without pathogenic B cell responses and production of autoantibodies. Clinical and demographic characteristics are summarized in table S1.

### Animal studies

All procedures in mice were in accordance with institutional guidelines and were reviewed and approved by the Greek Federal Veterinary Office (1044/1319; Athens, Greece). New Zealand black♀ × New Zealand white♂ F1 mice (i.e., NZB/W-F1) spontaneously develop an autoimmune syndrome resembling human SLE (*78*). NZB (NZB/OlaHsd) and NZW (NZW/OlaHsd) mice were purchased from Envigo. NZB/W-F1 were considered diseased when exhibiting ≥100 ng/dl of urine protein (following 6 months of life) and prediseased at 10 weeks old. Each experiment was repeated three times. All animals were maintained in the Biomedical Research Foundation Academy of Athens animal facility. All NZB/W-F1 mice used in the experiments were female. Mice were housed six per cage in a temperature- (21° to 23°C) and humidity-controlled colony room, maintained on a 12-hour light/12-hour dark cycle (07:00 to 19:00 light on), with standard food (4RF21, Mucedola Srl, Italy) and water provided ad libitum. All mice in the animal facility were screened regularly by a health-monitoring program, in accordance to the Federation of European Laboratory Animal Science Association and were free of pathogens.

### Proteomics

#### Sample preparation

B cells were isolated simultaneously (to avoid batch effects) from frozen [stored in 90% fetal bovine serum (FBS)/10% DMSO at −80°C] peripheral blood mononuclear cells (PBMCs) of patients with SLE and HC (*n* = 11 per condition) with the EasySep Human B Cell Isolation Kit (catalog no. 17954, STEMCELL Technologies), following deoxyribonuclease treatment of PBMCs. The purified cell population was subjected to complete cell lysis using a buffer consisting of 4% SDS, 100 mM tris/HCl, and 100 mM dithiothreitol (pH 7.6) and incubated at 95°C for 5 min. Lysed samples were further sonicated for 30 min in a water bath. The protein extracts were purified from debris by centrifugation for 20 min at 17,000*g*. Supernatants were transferred to clean tubes and processed according to the single-pot solid phase–enhanced sample preparation (SP3) method of Hughes *et al.* (*79*), without acidification and including a step of protein alkylation in 100 mM iodoacetamide. Digestion was carried out for continuous shaking at 1400 rpm at 37°C using 0.25-μg trypsin/Endoproteinase Lys-C from Lysobacter enzymogenes (LysC) mixture in a 25 mM ammonium bicarbonate buffer. The next day, the magnetic beads were removed, and the peptidic samples were further purified by SP3 peptide cleanup and evaporated to dryness in a vacuum centrifuge. The dried samples were solubilized in buffer A and sonicated for 5 min, and the peptide concentration was determined by measuring the absorbance at 280 nm using NanoDrop technology.

#### Ultrahigh pressure nanoLC

Each sample was analyzed three times (technical replicates). Approximately 0.5 μg of peptides was preconcentrated with a flow of 10 μl/min for 4 min using a C18 trap column (Acclaim PepMap100; 100 μm by 2 cm; Thermo Fisher Scientific) and then loaded onto a 50-cm-long C18 column (inside diameter, 75 μm; particle size, 2 μm; 100 Å; Acclaim PepMap100 RSLC, Thermo Fisher Scientific). The binary pumps of the high-performance liquid chromatography (RSLCnano, Thermo Fisher Scientific) consisted of solution A [2% (v/v) Acetonitrile (ACN) in 0.1% (v/v) formic acid] and solution B [80% (v/v) ACN in 0.1% (v/v) formic acid]. The peptides were separated using a linear gradient starting with 5% B up to 27.5% B in 58 min stepped to 40% B in 2 min and lastly reaching 99% B and remaining there for 5 min and then allowed to equilibrate for 20 min with a flow rate of 300 nl/min. The column was placed in an oven operating at 50°C.

#### Tandem mass spectrometry

The eluted peptides were ionized by a nanospray source and detected by a Q-Exactive HF-X mass spectrometer (Thermo Fisher Scientific, Waltham, MA, USA) operating in a data-dependent mode. The peptides were measured from 350 to 1500 mass/charge ratio (*m*/*z*) using a resolving power of 120,000 for MS1, Automatic Gain Control (AGC) at 3 × 10^6^, maximum injection time of 100 ms, followed by 12 tandem mass spectrometry of the most abundant 2^+^-4^+^ charged ions, using a resolving power of 15,000, AGC at 1 × 10^5^, maximum injection time of 22 ms, and an isolation window of 1.2 *m*/*z* at 28 Normalized Collision Energy (NCE) and a dynamic exclusion of 30 s. The software Xcalibur (Thermo Fisher Scientific) was used to control the system and acquire the raw files, and internal calibration was activated using a lock mass of *m*/*z* 445.12003.

#### Data analysis

The raw files were searched using the Proteome Discoverer 2.4, against the *Homo sapiens* reference proteome FASTA database downloaded from UniProt on 19 September 2019 (containing 95,959 protein sequences) and the ProteomeTools_HCD28_PD spectral library using the multiple peptide search option activated and using serially the MSPepSearch and SequestHT nodes. The protein dynamic modifications assessed were oxidation, +15.995 Da (M); deamidation, +0.984 Da (N, Q); and the N-terminal variable modifications of acetylation, +42.011 Da; Met-loss, −131.040 Da (M); and Met-loss + acetyl −89.030 Da (M). Carbamidomethyl/+57.021 Da (C) was set as a static modification. The result was filtered for high confident peptides, with enhanced peptide and protein annotations. Only master proteins were evaluated. The quantified abundances were based on intensity values and were normalized to the total peptide amount. The statistical evaluation between the protein-normalized abundances of the healthy individuals and patients with SLE was performed using the Proteome Discoverer software (pairwise background-based *t* test). The minimum percentage of replicate features was set to 60%. A total of 4995 proteins were identified with at least two peptides. The mass spectrometry proteomic data have been deposited to the ProteomeXchange Consortium via the PRIDE (*80*) partner repository with the dataset identifier PXD031389.

### Enrichment analysis of proteomic data

A list of 1094 differentially expressed proteins based on FDR (false discovery rate) < 0.05 and at least two peptides expressed were used as input for the enrichment analysis using STRING (*81*) and QIAGEN IPA (QIAGEN Inc.; https://digitalinsights.qiagen.com/IPA) (*82*) tools. FDR < 0.05 was used to determine significance for enriched Gene Ontology biological processes and Kyoto Encyclopedia of Genes and Genomes (KEGG) terms in STRING analysis and FDR < 0.2 to determine significance for enriched canonical pathways in IPA nondirectional analysis. The full lists of differentially expressed proteins and enriched terms are provided in table S2.

### RNA-seq data

Published data in fastq format (*15*) were downloaded and processed, as reported below. Quality of sequencing was assessed using FastQC software (*83*). Raw reads in fastq format were trimmed for adapter content and low-quality bases (*Q* < 30) at the 3′ end using cutadapt (*84*) and aligned to the human genome (hg38 version) using STAR 2.6 algorithm (*85*). Gene quantification was performed using HTSeq (*86*). GENCODE annotation file version 29 was used for the annotation. Differential expression analysis was performed using edgeR package in R (*87*).

#### Enrichment analysis of RNA-seq data

GSEA (*88*) was performed to reveal enriched signatures in our gene sets based on the Molecular Signatures Database v.7.0. Gene sets were ranked by taking the −log_10_ transform of the *P* value multiplied by the fold change. Significantly up-regulated genes were at the top, and significantly down-regulated genes were at the bottom of the ranked list. GSEA preranked analysis was then performed using the default settings. Enrichment was considered significant when FDR (*q* value) < 25%.

### Human cell isolation from peripheral blood

Heparinized blood (10 ml) was collected from healthy subjects and individuals with SLE or AS. PBMCs were isolated on Histopaque-1077 (Sigma-Aldrich) density gradient. Briefly, blood was diluted 1:1 with phosphate-buffered saline (PBS) and layered over Histopaque-1077 solution (Sigma-Aldrich). Tubes were centrifuged at 500*g* for 30 min with no brake at room temperature. PBMC layer was collected, and cells were washed with PBS. In case neutrophils were needed in addition to PBMCs, cell isolation was done on double gradient of Histopaque-1077/Histopaque-1119 (Sigma-Aldrich). Briefly, blood was diluted 1:2 with PBS and layered over Histopaque-1077/Histopaque-1119 (1:1). Tubes were centrifuged at 1200*g* for 30 min with no brake at room temperature. Neutrophils were collected at the interface of the Histopaque-1119 and Histopaque-1077 layers and washed with PBS. For the isolation of total B cells, the EasySep Human B Cell Isolation Kit (catalog no. 17954, STEMCELL Technologies) was also used in most experiments following the Histopaque-1077 protocol.

### Flow cytometry and cell sorting

For analysis and isolation of immune cells, single-cell suspensions from fresh human PBMCs or neutrophils were stained with conjugated antibodies against: CD19, CD4, CD8, CD25, HLA-DR, CD14, CD16, and CD66b. All sorted cells were 7-AAD negative (420404, BioLegend). For the intracellular staining including Foxp3, γΗ2ΑΧ (Ser^139^), Κi67, pATM (Ser^1981^), and cPARP1 (Asp^214^), cells were fixed and stained using the Foxp3 Staining Set (catalog no. 00-5523-00, eBioscience) according to the manufacturer’s instructions. Cells were sorted on a FACSAria III (BD Biosciences) using the BD FACSDiva v8.0.1 software (BD Biosciences). For analysis of cultured cells following EasySep Human B Cell Isolation, cells were stained with conjugated antibodies against CD19, CD40, CD80, BAFF, HLA-DR, IgD, CD27, CD38, IgM, and IgG. For apoptosis detection upon administration of DDR pharmaceutical inhibitors, cultured B cells were further stained with anti-CD19, annexin V (catalog no. 640908, BioLegend), and 7-AAD according to the manufacturer’s recommendations. For analysis of peripheral B cells from mice, PBMCs were isolated from blood on Histopaque-1077 density gradient. Then, PBMCs were stained with conjugated antibody against B220/CD45R and γΗ2ΑΧ (Ser^139^). Data acquisition was performed on a FACSAria III (BD Biosciences), BD FACSCelesta, and the BD FACSDiva v8.0.1 software (BD Biosciences). Analysis was performed with FlowJo software. Table S3 includes details on the antibodies (fluorochrome, clone, vendor, catalog number, and concentration).

### Cell cycle assessment

For the cell cycle analysis via flow cytometry, 200,000 to 500,000 B cells per sample (following EasySep Human B Cell Isolation from PBMCs) were first stained extracellularly with anti-CD19 antibody in 200 μl of 5% FBS/PBS buffer for 10 min at room temperature to ensure B cell purity, and then, following washing with PBS, cells were fixed and stained for Ki67 and γΗ2ΑΧ using the Foxp3 Staining Set according to the manufacturer’s instructions. At the end, cells were stained with 7-AAD cellular DNA content marker (5 μl per sample) in 200 μl of 5% FBS/PBS buffer for 15 min at room temperature, washed with PBS, and then resuspended in 300 μl of 5% FBS/PBS. Cells were analyzed using BD FACSCelesta using the BD FACSDiva v8.0.1 software. Linear scale was used for 7-AAD. This method allows the assessment of γΗ2ΑΧ expression across apoptosis, G_0_, G_1_, S, and G_2_-M phases. For the analysis of B cells upon ATRi, B cells were cultured for 3 days with IFN-α (850 U/ml), IL-21 (50 ng/ml)/CpGb (2.5 μg/ml)/sCD40L (1 μg/ml) survival, and mild proliferation stimuli in the presence or absence of ATRi (2 μΜ) or DMSO (control) in P96 round wells (200,000 cells per well) and then stained and analyzed as mentioned above.

For the cell cycle analysis via confocal microscopy, B cells were cultured for 45 hours under the following conditions: (i) SLE B cells with IL-21/CpGb/sCD40L cocktail for survival and mild induction of proliferation, as described above, (ii) HC B cells treated with IFN-α and IL-21/CpGb/sCD40L cocktail (SLE-like B cells), and (iii) HC B cells with IL-21/CpGb/sCD40L cocktail (control condition). After 45 hours of culture, cells were exposed to an additional 3 hours of culture with EdU (5 μΜ) to capture cells being in S phase of the cell cycle, and then, following EdU wash-off, we proceeded with the Click-iT EdU Cell Proliferation protocol for Imaging (catalog no. C10337, Thermo Fisher Scientific) along with simultaneous staining of pH3 (1:100; for detection of G_2_-M mitotic cells), pATR (Thr^1989^; 1:100), and Hoechst 33342 (nuclear counterstain). At least 80 cells per human subject were analyzed. The secondary antibodies were Alexa Fluor 555 anti-mouse IgG (1/500) for pH3 and Alexa Fluor 647 anti-rabbit IgG (1/200) for pATR (table S3).

### Immunofluorescence and confocal microscopy

For the cell immunofluorescence experiments, cells (isolated by either cell sorting or the EasySep Human B Cell Isolation Kit) were seeded in coverslips pretreated with poly-l-lysine, fixed with 4% paraformaldehyde for 15 min at room temperature, and washed twice with PBS. Cells were blocked and permeabilized with 1% bovine serum albumin dissolved in PBS containing 0.1% Triton X-100 (blocking buffer) for 30 min at room temperature. Next, cell-seeded slides were incubated with primary antibodies and 4′,6-diamidino-2-phenylindole (DAPI) in blocking buffer at room temperature for 1 hour, followed by three washes with PBS containing 0.1% Triton X-100 and then by secondary antibodies for 45 min at room temperature in the dark. Last, cells were mounted with ProLong Diamond Antifade Mountant (catalog no. P36961, Thermo Fisher Scientific) and visualized using inverted or upright confocal imaging system Leica SP5. Puncta/cell or intensity/cell was calculated using a macro developed in Fiji software (*89*). The primary antibodies in the immunofluorescence were against γΗ2ΑΧ (Ser^139^; 1/200), pATR (Thr^1989^; 1/100), pChk1 (Ser^345^; 1/50), p-p53 (Ser^15^; 1/100), pChk2 (Thr^68^; 1.2/100), p95/NBS1 (1/100), cleaved caspase-3 (Asp^175^; 1/800), and pDNA-PKcs (Ser^2056^; 1/150), and the secondary antibodies were Alexa Fluor 555 anti-mouse IgG (1/500), Alexa Fluor 488 anti-rabbit IgG (1/500), and CF 555 anti-rabbit IgG (1/1000; table S3). For the analyses, 60 to 100 cells per human subject (corresponding to a minimum of four different fields of the coverslip) were observed for each marker under confocal microscopy.

### Cell culture and chemical inhibition

B cells from healthy individuals were cultured following isolation from fresh PBMCs (EasySep Human B Cell Isolation Kit) in a 37°C humidified incubator with 5% CO_2_ while plated in a 96-well round-bottom plate (Sarstedt) in a concentration of 1.5 × 10^5^ cells per well. Cells were cultured in RPMI 1640 with GlutaMAX (catalog no. 61870036, Gibco) supplemented with 10% (v/v) heat-inactivated FBS (catalog no. 10270106, Gibco), penicillin-streptomycin (100 U/ml and 100 μg/ml, respectively; catalog no. 15140m, Gibco), sodium pyruvate (1 mM; catalog no. 11360070, Gibco), Hepes (10 mM; catalog no.15630106, Gibco), and 2-mercaptoethanol (0.05 mM; catalog no. 31350010, Gibco). In addition, all cultured B cells, irrespective of experiment, were supplemented with a “survival/mild proliferation” cocktail of IL-21 (50 ng/ml; catalog no. 200-21, PeproTech), CpG-B (2.5 μg/ml; catalog no. HC4039, Hycult Biotech), and sCD40L/CD154 (1 μg/ml; catalog no. 11343345, ImmunoTools). To mimic DDR in SLE B cells, IFN-α (catalog no. 11200-1, PBL Assay Science) was added to cells at 850 U/ml. For chemical ATR inhibition, berzosertib (i.e., ATRi; catalog no. S7102, Selleckchem) was added to cells at 2 or 5 μΜ, and for Chk1 inhibition, CHIR-124 (i.e., Chk1i; catalog no. HY-13263, Selleckchem) was added to cells at 50 nM (details on legends).

### Measurement of immunoglobulins and cytokines

Released immunoglobulins were measured with the LEGENDplex Human Immunoglobulin Isotyping Panel (8-plex) according to the manufacturer’s instructions, following the collection of supernatants at day 7 from cultured B cells treated with IFN-α (850 U/ml) in the presence or absence of ATRi (2 μΜ). Released cytokines were measured with LEGENDplex Human B Cell Panel (13-plex) following the collection of supernatants at day 2 from cultured B cells treated with IFN-α (850 U/ml) in the presence or absence of ATRi (5 μΜ). Data acquisition was done on FACSAria III (BD Biosciences) and the BD FACSDiva v8.0.1 software (BD Biosciences). Analysis was performed with the LEGENDplex Data Analysis Software.

### IRF1 knockdown assay

For IRF1 gene knockdown, Silencer Select siRNA (i.e., siIRF1) was used (100 nM; catalog no. 4392420, Ambion), and Silencer Select Negative Control siRNA (i.e., siNeg) was used as a control (100 nM; catalog no. 4390843, Ambion). Cells were transfected with the small interfering RNAs (siRNAs) using Lipofectamine 2000 (catalog no. 11668019, Invitrogen) according to the manufacturer’s protocol. Briefly, 3 × 10^5^ cells were plated per well in a 96-well flat-bottom plate in Opti-MEM I Reduced Serum Medium (100 μl per well; catalog no. 31985070, Gibco), and the appropriate siRNAs with the transfection reagents were added for 5 hours. After 5 hours, the supernatant was discarded, and the aforementioned supplemented medium and survival/mild proliferation cocktail were added (*V*_final_ = 200 μλ per well). After 42 hours from the initial plating, IFN-α (850 U/ml) was added to all wells. For gene expression analysis, RNA was isolated 2 days after the initial plating; for protein expression analysis, cells were collected 4 days after the initial plating, and for cell subsets and proliferation assays, cells were collected 5 days after the initial plating.

### ChIP experiment

To analyze the molecular interactions of ATR in B cells upon IFN-α stimulation, ChIP experiment was carried out. Briefly, 4 × 10^5^ cells were plated per well in a 96-well round-bottom plate in medium with survival/mild proliferation cocktail and IFN-α. Cells were collected 6 hours after culture with the aforementioned reagents, and ChIP was performed with the Magna ChIP A/G Chromatin Immunoprecipitation Kit (catalog no. 17-10085, Merck) according to the manufacturer’s protocol. The antibodies used were against IRF1 (catalog no. 8478, Cell Signaling) and control IgG (catalog no. 3900, Cell Signaling Technology; table S3). Detection and analysis of ChIP precipitates were performed by real-time quantitative polymerase chain reaction (qPCR) using primers for the promoter region of *ATR*. In all cases, data (*C*_t_ values) derived from the input sample were used for normalization by the “percent of input (%IP)” method and presented as fold of change relative to control anti-IgG IPs. The sequences of the core consensus response element for IRF1 were identified on *ATR* promoter sequence using the Gene Transcription Regulation Database (*90*) and EPD (*91*). The primers sequences are as follows: *ATR* loc1-forward, 5′-TGCTGTAATCTTGTGAGGTAGACA-3′; *ATR* loc1-reverse, 5′-GGGATTGGGAGTTACAGGCC-3′; ATR loc2-forward, 5′-TCTTGCTTCTCTGTGCCTCC-3′; *ATR* loc2-reverse, 5′-GGCTTCTTTCTCAGCACCGA-3′; *ATR* loc3-forward, 5′-CACTAGTCAACCACGCCAAC-3′; and *ATR* loc3-reverse, 5′-CCCGGGTCCTATGCAGAAAA-3′.

### Quantitative PCR analysis (real-time RT-qPCR)

Cells were lysed in buffer RA1 (Macherey-Nagel), and RNA was extracted using a NucleoSpin RNA XS isolation kit according to the manufacturer’s instructions. First-strand complementary DNA synthesis was performed using the PrimeScript RT-PCR Kit (catalog no. RR037A, Takara). qPCR was carried out using the KAPA SYBR Fast Universal Kit (catalog no. KK4602, Kapa Biosystems). Relative expression of target genes was calculated by comparing them to the expression of the housekeeping genes *ACTINB* or glyceraldehyde phosphate dehydrogenase (*GAPDH*).

The primers sequences that were used for real-time reverse transcription qPCR (RT-qPCR) are the following: *ATR* forward, 5′-GGAGATTTCCTGAGCATGTTCGG-3′; *ATR* reverse, 5′-GGCTTCTTTACTCCAGACCAATC-3′; *ATM* forward, 5′-TGTTCCAGGACACGAAGGGAGA-3′; *ATM* reverse, 5′-CAGGGTTCTCAGCACTATGGGA-3′; *IRF1* forward, 5′-CCAAGAGGAAGTCATGTG-3′; *IRF1* reverse, 5′-TAGCCTGGAACTGTGTAG-3′; ACTINB forward, 5′-CTCTTCCAGCCTTCCTTCCT-3′; *ACTINB* reverse, 5′-AGCACTGTGTTGGCGTACAG-3′; *GAPDH* forward, 5′-GCACCACCAACTGCTTAG-3′; and *GAPDH* reverse, 5′-GCCATCCACAGTCTTCTG-3′.

### Western blotting

Whole-cell extracts were lysed with vortex (vortex every 5 min for 20 min with 1-min breaks in between, in ice) in radioimmunoprecipitation assay mix [150 mM sodium chloride, 50 mM tris-HCl (pH 8.0), 1% Nonidet P-40, 0.5% sodium deoxycholate, and 0.1% SDS] supplemented with protease (1×; catalog no. 1183617001, Roche) and phosphatase (1:100; catalog no. P5726, Sigma-Aldrich) inhibitor; then, they were centrifuged at full speed for 10 min at 4°C, and the supernatant was collected (protein lysate). Protein amounts were determined using detergent compatible (DC) protein assay (catalog no. 5000112, Bio-Rad) according to the manufacturer’s instruction. β-Mercaptoethanol (6×) was added to the samples, and they were heated for 5 min at 95°C. Cell extracts were resolved using 4 to 20% precast polyacrylamide gel (catalog no. 4561094, Bio-Rad) and transferred to nitrocellulose membrane using a transfer apparatus according to the manufacturer’s instructions (Bio-Rad). The protein loading amount per well was determined to 20 μg. Membranes were saturated with 5% nonfat milk diluted in tris-buffered saline–0.1% Tween 20 for 1 hour and incubated with primary antibodies overnight at 4°C and with anti–mouse–horseradish peroxidase (HRP) or anti–rabbit-HRP secondary antibodies for 1 hour. Blots were developed with enhanced chemiluminescence (catalog no. 34580, Thermo Fisher Scientific) according to the manufacturer’s instructions. For the analysis, quantification of phosphoprotein expression has been performed by using the normalization with the corresponding total protein: Both phosphorylated and total proteins were first normalized with housekeeping protein expression. Antibodies are depicted in table S3.

### Statistical analysis

Statistical analysis was performed taking into account the experimental setup using paired or unpaired Student’s *t* test and one-way or two-way analysis of variance (ANOVA) in GraphPad Prism v8.0.1 software, as indicated in the figure legends. Data are presented as means ± SEM. *P* < 0.05 was considered as indicative of statistical significance. All *P* values and *n* are reported in the figure legends. The investigators were not blinded to the identities of the samples. Compared samples were collected and analyzed under the same conditions. Each experiment was repeated at least three times. For LEGENDplex experiments, it was one run per assay composed of three independent experiments where samples were collected.

## Notes

View/request a protocol for this paper from *Bio-protocol*.
