# Supplementary material for: ATR-mediated DNA damage responses underlie aberrant B cell activity in systemic lupus erythematosus
Source: Sci Adv. 2022 Oct 28;8(43):eabo5840. doi: 10.1126/sciadv.abo5840 (PMC9616496; doi:10.1126/sciadv.abo5840)
Supplement: Supplementary file 1 — Figs. S1 to S16 [file sciadv.abo5840_sm.pdf]

Science Advances
AAAS

Supplementary Materials for

ATR-mediated DNA damage responses underlie aberrant B cell activity in systemic lupus erythematosus

Theodora Manolakou *et al.*

Corresponding author: Theodora Manolakou, dmanolakou@bioacademy.gr; Panayotis Verginis, pverginis@bioacademy.gr; Dimitrios T. Boumpas, boumpasd@uoc.gr

Sci. Adv. **8**, eabo5840 (2022)
DOI: 10.1126/sciadv.abo5840

The PDF file includes:

Figs. S1 to S16
Legends for tables S1 to S3

Other Supplementary Material for this manuscript includes the following:

Tables S1 to S3

Figure S1

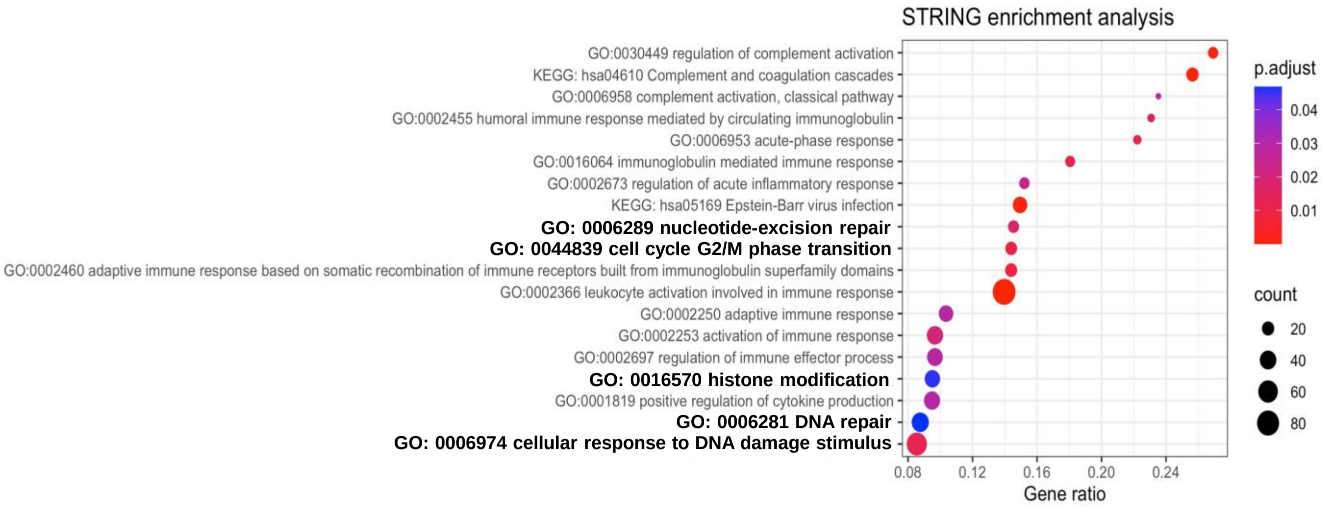

Fig. S1. STRING enrichment analysis reveals enriched DDR of human SLE B cells. STRING enrichment analysis was performed using the 1094 differentially expressed proteins (FDR<0.05 & at least 2 peptides expressed) between SLE and HC (n=11 per group). Selected enriched gene ontology (GO) biological processes (BP) and KEGG pathways (FDR<0.2) are shown. DDR-related pathways are indicated in bold.

Figure S2

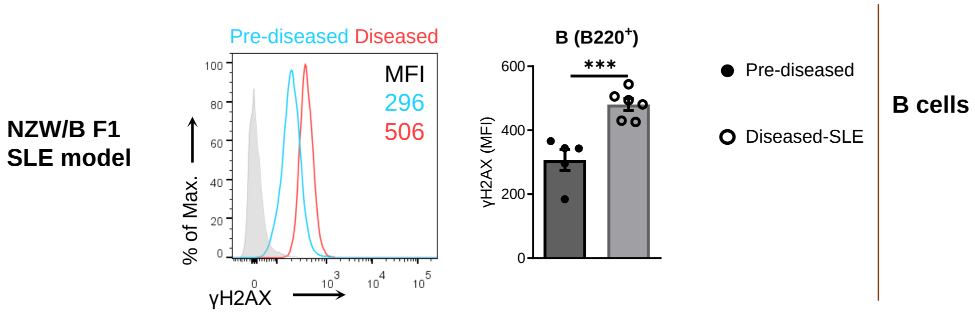

Fig. S2. Increased DDR in murine SLE B cells. PBMCs were isolated from the peripheral blood of NZB/W F1 SLE diseased (n=5) and pre-diseased (n=6) mice and were stained for B220 and γH2AX DNA damage detection marker. Then they were analyzed through flow cytometry. Representative plot of MFI showing overlay of unstained cells (grey), stained diseased cells (red) and stained pre-diseased cells (light blue) is depicted. MFI: mean fluorescent intensity. Results are expressed as mean ± SEM. Unpaired Student's t test, *** $p<0.001$.

Figure S3

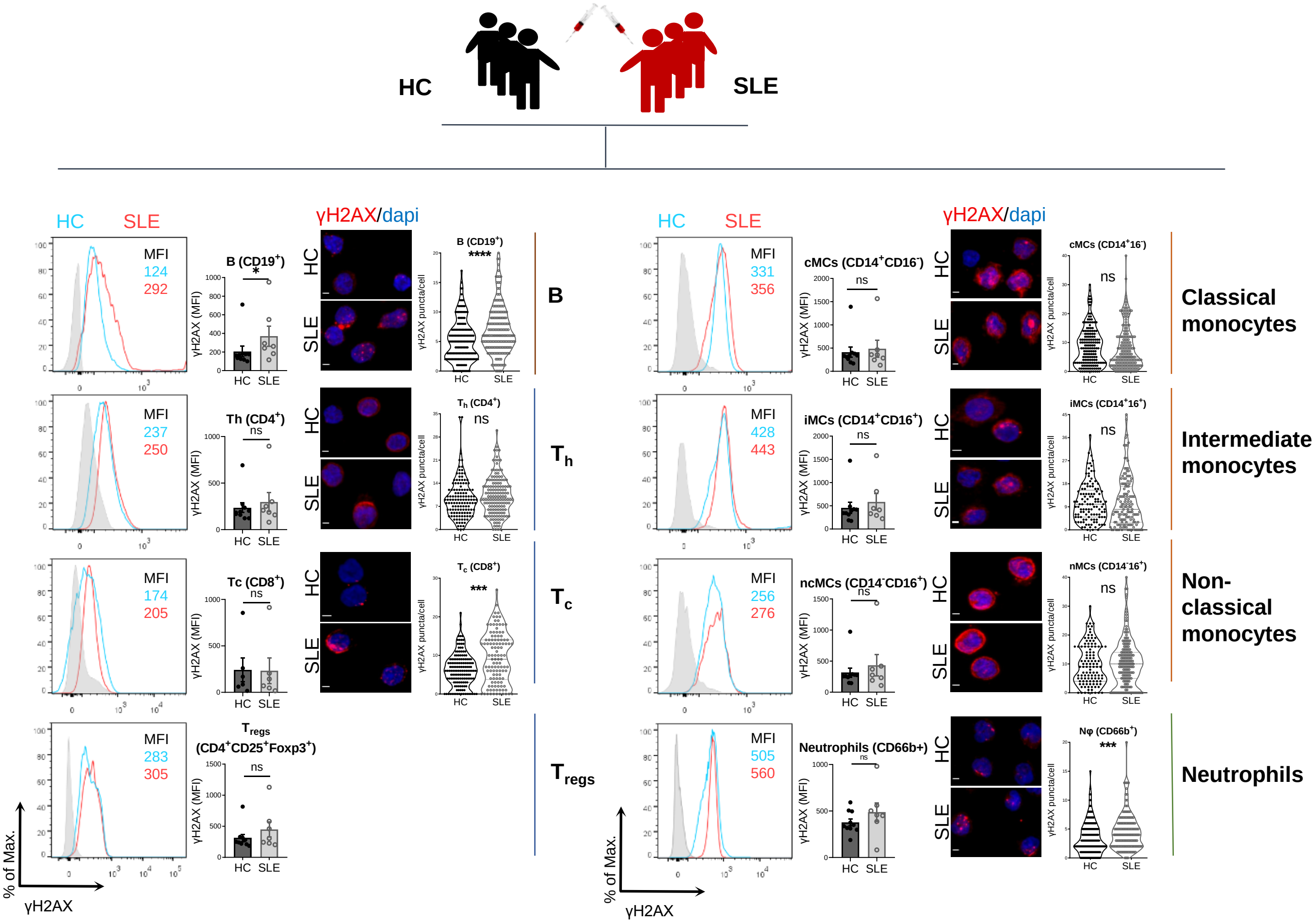

Fig. S3. DNA damage investigation in peripheral immune cells in SLE highlights B cells. Screening of peripheral blood immune cells of SLE compared to healthy controls (HC) for DNA damage detection: Expression of γH2AX DNA damage detection marker in B ($CD19^+$), T helper ($CD4^+$), T cytotoxic ($CD8^+$), T regulatory ($CD4^+CD25^+Foxp3^+$), classical monocytes ($HLA\text{-}DR^+CD14^+CD16^-$), intermediated monocytes ($HLA\text{-}DR^+CD14^+CD16^+$), non-classical monocytes ($HLA\text{-}DR^+CD14^-CD16^+$) and neutrophils ($CD66b^+$) cells of SLE patients compared to HC via flow cytometry (SLE; n=7 or 6, HC; n=10 or 6) and/or confocal microscopy (FACS-sorted cells; SLE: n=2-3, HC: n=3). Representative histograms showing overlay of unstained cells (grey), stained SLE cells (red) and stained HC cells (light blue), and immunofluorescence confocal microscopy images. Cells on confocal are labeled with DAPI nuclear staining (blue). Scale bar: 2μM. Results are expressed as mean ± SEM. Unpaired Student's t test was applied, $p \geq 0.05$ (ns), * $p<0.05$, ** $p<0.01$, *** $p<0.001$, **** $p<0.0001$. ES: enrichment score, HC: healthy controls, Th: T helper, Tc: T cytotoxic, Tregs: T regulatory, cMCs: classical monocytes, iMCs: intermediate monocytes, ncMCs: non-classical monocytes, Nφ: neutrophils, MFI: mean fluorescent intensity.

Figure S4

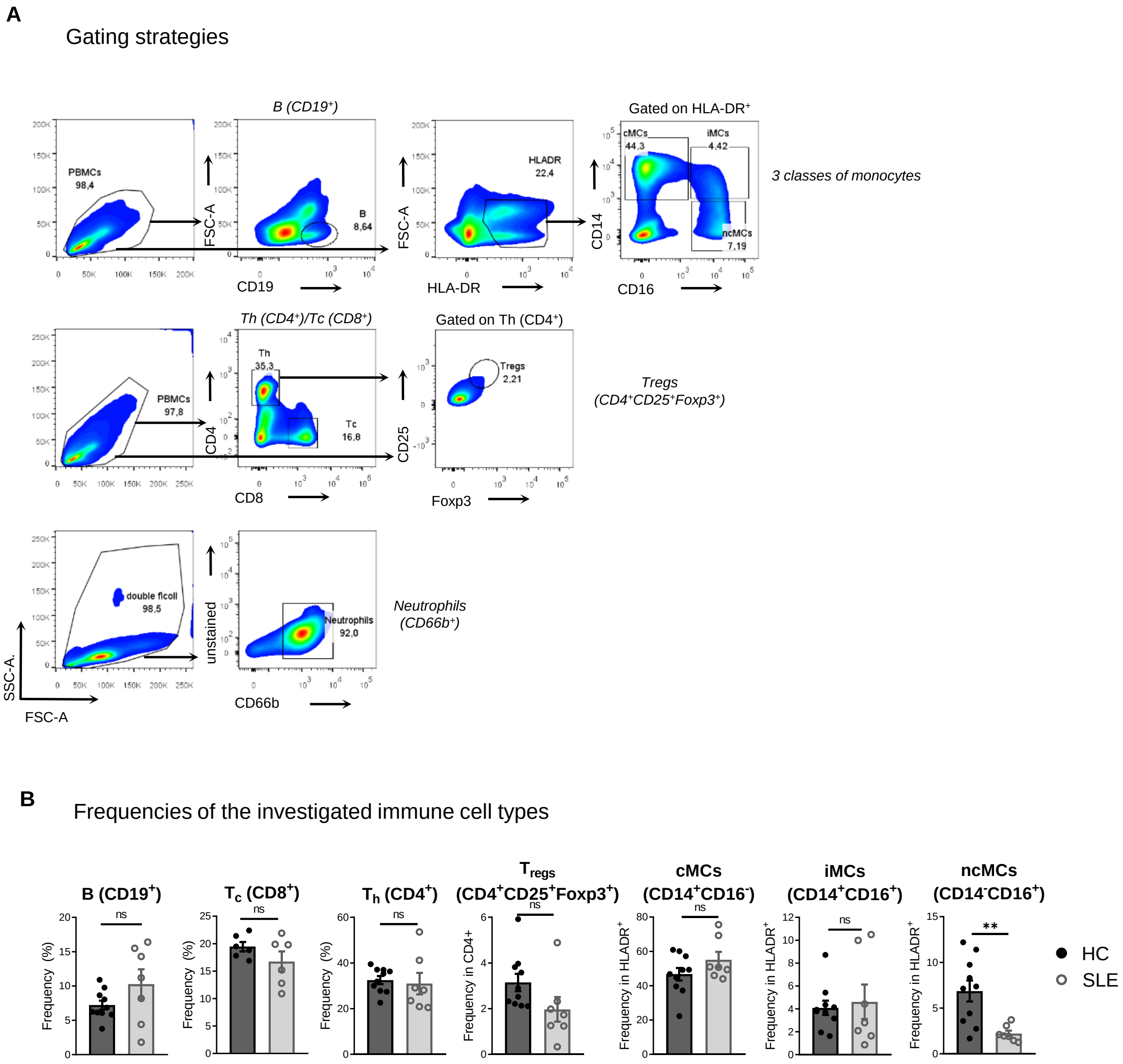

Fig. S4. Representative flow cytometry gating strategy for identification of immune cells. (A) Gating strategies for the FACS-sorted (for confocal) and/or flow cytometry-analyzed immune cells of the samples shown in Fig. S3 for γH2AX marker, following PBMCs or neutrophils isolation from the peripheral blood. **(B)** Immune cell frequencies of the samples shown in Fig. S3 (SLE; n=7 or 6, HC; n=10 or 6). Results are expressed as mean ± SEM. Unpaired Student's t test, $p \geq 0.05$ (ns), ** $p < 0.01$.

Figure S5

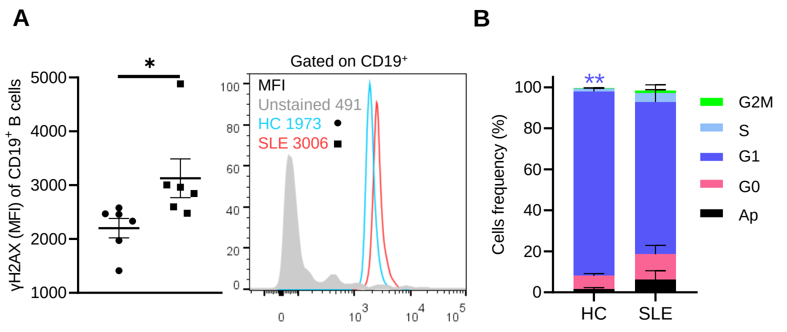

Fig. S5. Cell cycle phases of SLE and HC B cells. (A) B cells were isolated from the peripheral blood of SLE patients and HC (n=6 per group) using magnetic bead-based approach and analyzed for intracellular γH2AX expression via flow cytometry gated on $CD19^{+}$ B cells. Unstained sample was used as negative control. *$p<0.05$ (unpaired Student's t test). **(B)** Cell cycle analysis using Ki67 and 7-AAD via flow cytometry. The comparison was done between the same cycle phase of the two conditions (SLE and HC). Only G1 phase -derived cells exhibited statistical significance, **$p<0.01$ (two-way ANOVA). Representative flow cytometry plots are presented in Figure 1E.

Figure S6

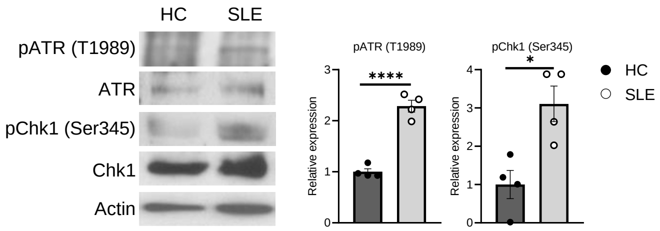

Fig. S6. Activation of ATR pathway in SLE B cells as shown via western blot. B cells were isolated from SLE patients and HC (n=4 per group) using magnetic bead-based approach and were used for western blot analysis with pATR (T1989), ATR, pChk1 (Ser345) and Chk1 antibodies. Actin blotting was used to confirm equal loading of each sample. Quantification of pATR and pChk1 expression has been performed by utilizing the normalization with the total ATR and Chk1 protein, respectively. Both phospho- and total proteins were normalized with actin expression. Representative samples depicted. Results are presented as mean ± SEM. *p<0.05, **** p<0.0001 (unpaired Student's t test).

Figure S7

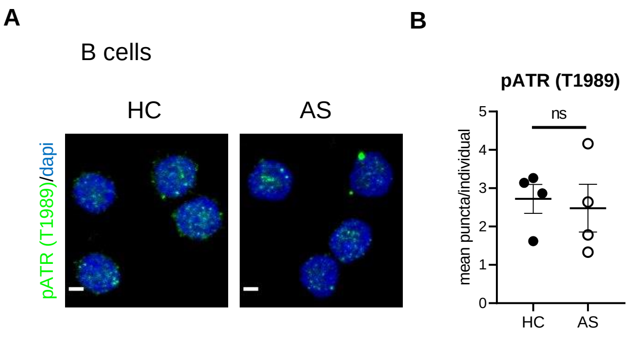

Fig. S7. ATR is not activated in AS B cells. B cells were isolated using magnetic bead-based approach from the peripheral blood of patients with ankylosing spondylitis (AS) and healthy controls (HC) (n=4 per group). **(A)** Representative images of B cells from individuals with AS or HC stained with anti-pATR (Thr1989) antibody and then with a fluorochrome-conjugated secondary antibody (green), were captured by confocal microscopy. Nuclei were stained with DAPI (blue). Scale bar: 2μM. **(B)** Images of B cells analyzed for pATR staining as mean puncta/cell per individual via fiji software. Results are expressed as mean ± SEM. Unpaired Student’s t test was applied, $p \geq 0.05$ (ns).

Figure S8

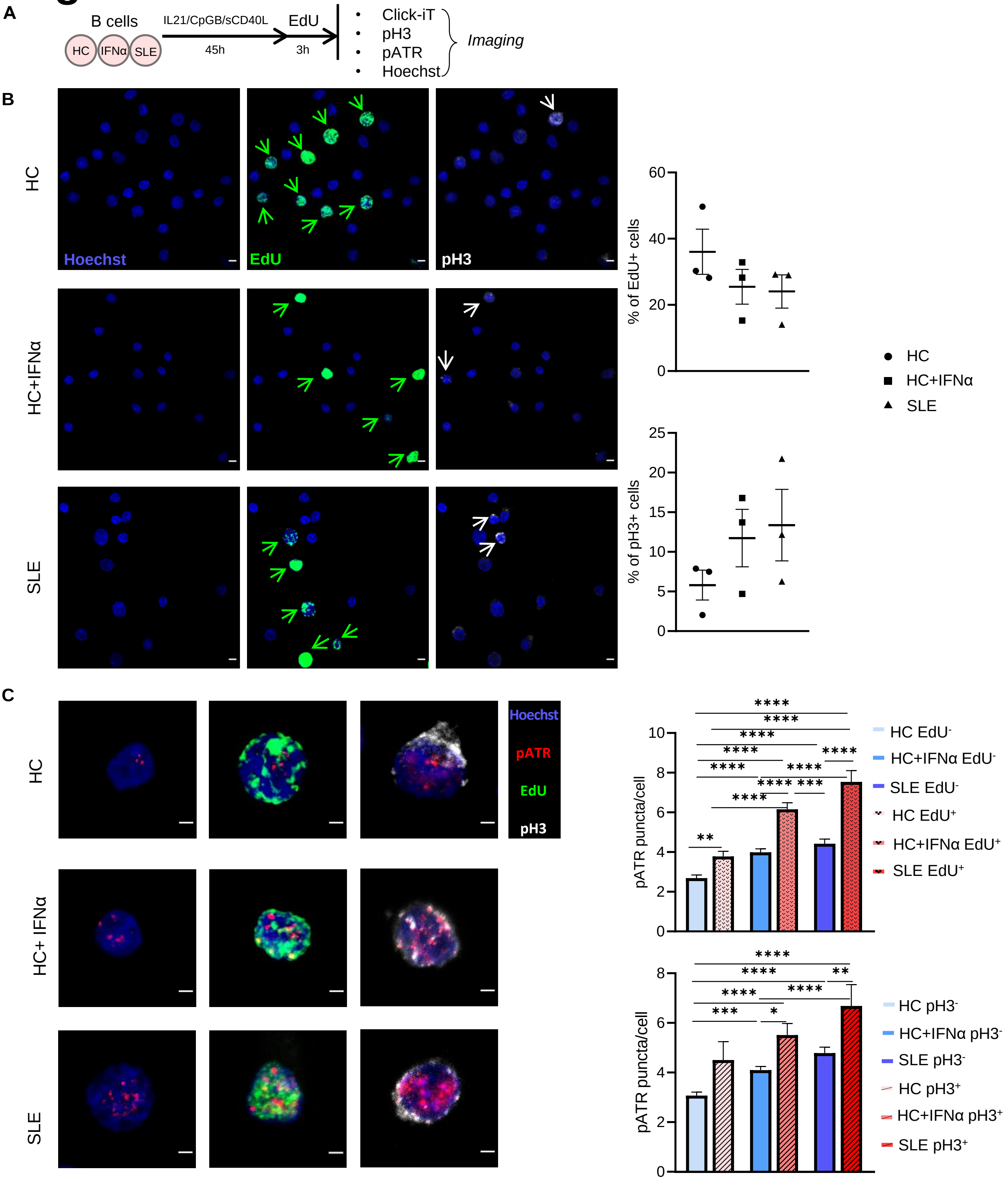

Fig. S8. Increased pATR in replicative and mitotic SLE B cells. (A) B cells were isolated from HC and SLE patients (n=3 per group) using magnetic bead-based approach and were cultured for 45h with IL21/ CpGb/ sCD40L survival and mild proliferation stimuli in the presence or absence of IFNα (850U/ml) (in the case of HC), followed by 3 hours of EdU treatment and immunofluorescence staining as indicated in the schematic representation. **(B)** Representative confocal microscopy images and quantification of replicative (EdU$^+$) and mitotic (pH3$^+$) HC, HC+IFNα and SLE B cells. No statistical significance was observed (one-way ANOVA). **(C)** Representative confocal microscopy images and quantification of pATR in EdU$^-$, EdU$^+$, pH3$^-$ and pH3$^+$ cells from HC, HC treated withIFNα and SLE B cells. Analysis for pATR staining was performed as puncta per cell using a macro developed in Fiji software (2). *p<0.05, ** p<0.01, *** p<0.001, **** p<0.0001 (one-way ANOVA). Scale bar: 2μm. Results are presented as mean ± SEM.

Figure S9

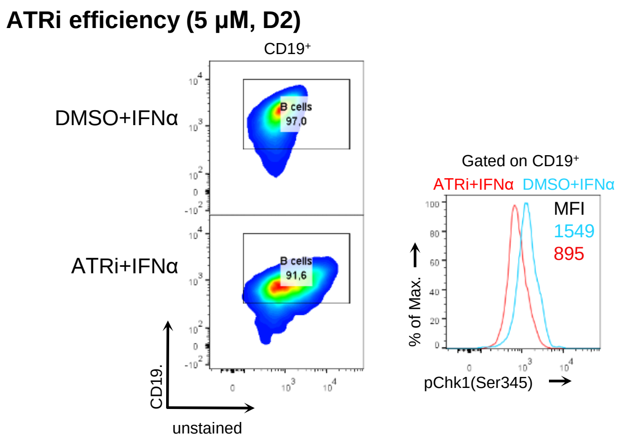

Fig. S9. ATRi efficiency. Efficiency of ATRi (berzosertib) is demonstrated by the reduced levels pChk1 (Ser345) in IFNA-treated B cells as depicted in the histogram.

Figure S10

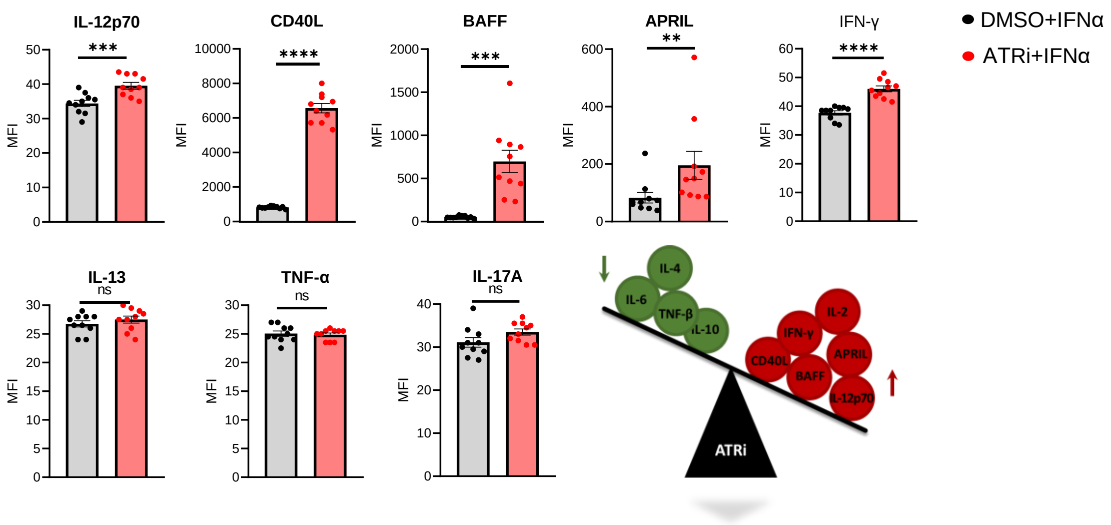

Fig. S10. ATRi alters release of cytokines in IFNα-treated B cells. Detection of released cytokines (TNF-α, IFN-γ, IL-17A, IL-12p70, APRIL, BAFF, CD40L) involved in B cell function, activation, proliferation and survival utilizing LEGENDplex™ technology through flow cytometry at day 2 (D2) of culture (n=10 individuals per condition) in the presence or absence of ATRi (5µM) or DMSO (control). Graphical representation of the overall results including the cytokines of Fig.3F (green: downregulated, red: upregulated). Results are expressed as mean ± SEM. Paired Student's t test, $p \geq 0.05$ (ns), *$p<0.05$, ** $p<0.01$, *** $p<0.001$, **** $p<0.0001$. MFI: mean fluorescent intensity.

Figure S11

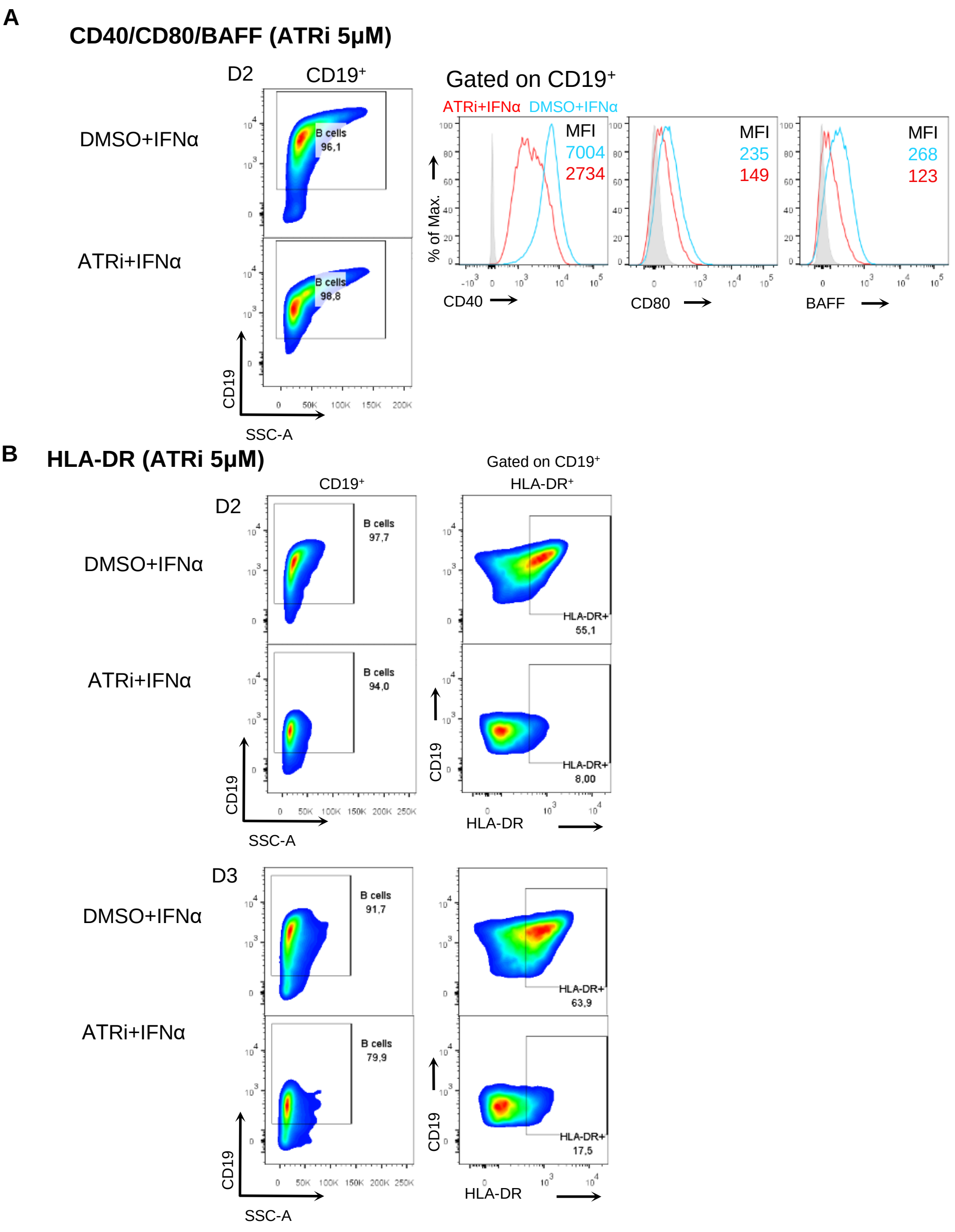

Fig. S11. ATRi inhibits activation and antigen presentation in IFNα-treated B cells. B cells were isolated from the peripheral blood of healthy individuals using magnetic bead-based approach, cultured with Il21/ CpGB/ sCD40L survival and mild proliferation stimuli cocktail in the presence of IFNα (850 U/ml) and in the presence or absence of ATRi or DMSO (control), and then stained for extracellular activation and antigen-presentation markers for flow cytometry assays **(A)** Representative gating strategies and histograms for CD40/CD80/BAFF for Fig. 4. **(B)** Representative gating strategies for HLA-DR for Fig.4.

Figure S12

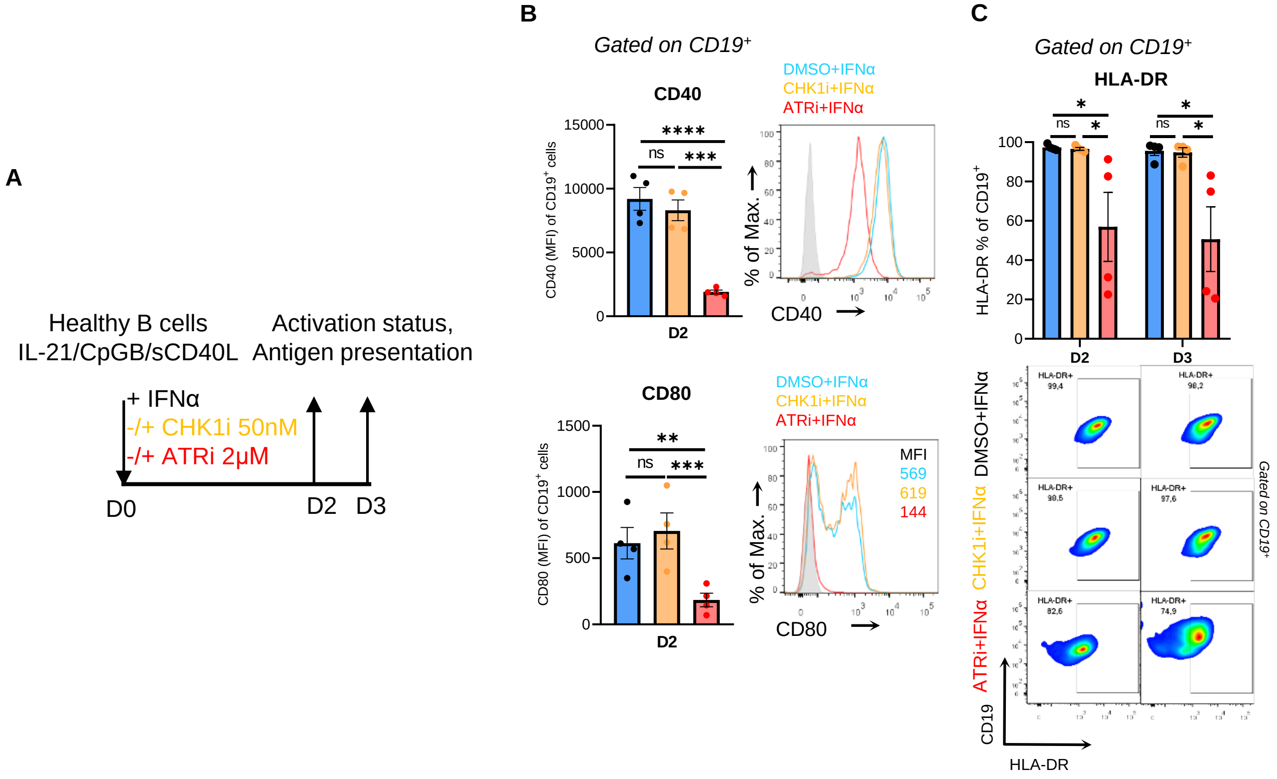

Fig. S12. ATRi inhibits activation in IFNα-treated B cells even when administrated in a low dose and this effect is specific of ATR perturbation and not of downstream Chk1. (A) Schematic representation of ATRi (2µM) *and CHK1i (50nM)* experiment at IFNα-treated B cells *ex vivo* for assessing cell activation status. Quantification of flow cytometry retrieved data for **(B)** CD40 (n=4 individuals per condition), CD80 (n=4 individuals per condition) and **(C)** HLA-DR (n=4 individuals per condition). Representative histograms showing overlay of unstained cells (grey), stained DMSO+IFNα treated cells (light blue), stained CHK1i+IFNα treated cells (light orange) and stained ATRi+IFNα treated cells (red). Results are expressed as mean ± SEM. Paired Student's t test, $p \geq 0.05$ (ns), * $p<0.05$, ** $p<0.01$, *** $p<0.001$, **** $p<0.0001$.

Figure S13

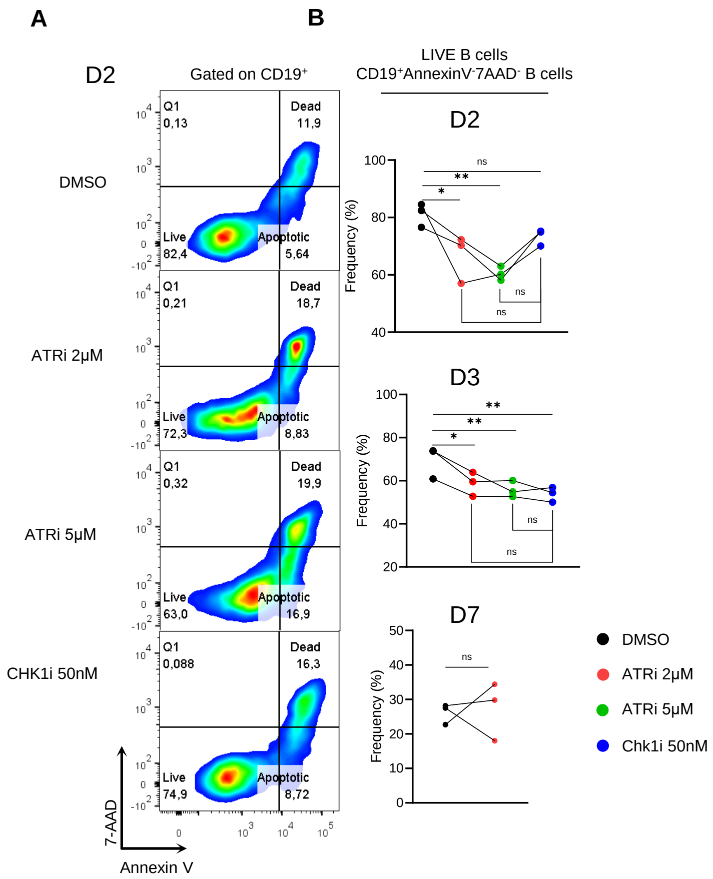

Figure S13. Evaluation of ATRi toxicity in B cells. (A) Representative flow cytometry plots for assessing live (CD19$^+$AnnexinV$^-$7AAD$^-$), apoptotic (CD19$^+$AnnexinV$^+$7-AAD$^-$) and dead (CD19$^+$AnnexinV$^+$/7AAD$^+$) B cells according to CD19, 7-AAD and Annexin V staining of isolated B cells upon DMSO, ATRi 2 µM, ATRi 5 µM and CHK1i 50 nM at day 2 (D2) of culture. All cultured B cells have been subjected to IFNα (850 U/ml) and IL-21/CpGB/sCD40L. **(B)** Viability analysis of B cells based on (A). N=3 individuals per condition. One-way repeated measures ANOVA. $p \geq 0.05$ (ns), *$p<0.05$, ** $p<0.01$, *** $p<0.001$, **** $p<0.0001$

Figure S14

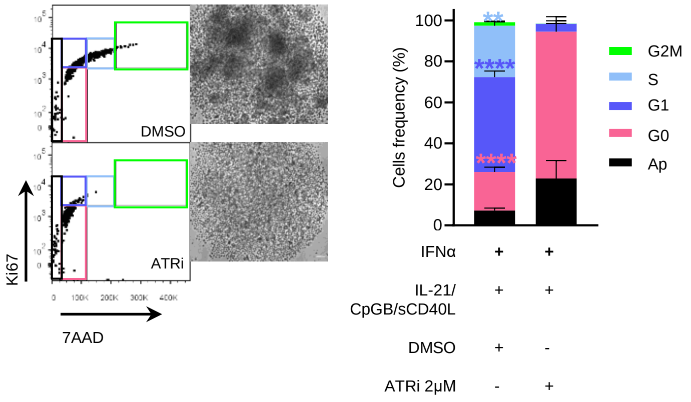

Fig. S14. ATRi affects cell cycle progression of SLE-like B cells. B cells were isolated from healthy individuals (n=3) using magnetic bead-based approach and were cultured for 3 days with IFNα (850U/ml), IL21/ CpGb/ sCD40L survival and mild proliferation stimuli in the presence or absence of ATRi (2μM) or DMSO (control), followed by Ki67/7-AAD cell cycle analysis via flow cytometry. Linear scale was used for 7-AAD (DNA content). The comparison was done between the same cycle phase of the two conditions (ATRi and DMSO). G0, G1 and S phase -derived cells presented statistical significance between the two conditions. Representative flow cytometry plots and corresponding microscopy images. ** $p<0.01$, **** $p<0.0001$ (two-way ANOVA). Results are presented as mean ± SEM. Scale bar: 10μM.

Figure S15

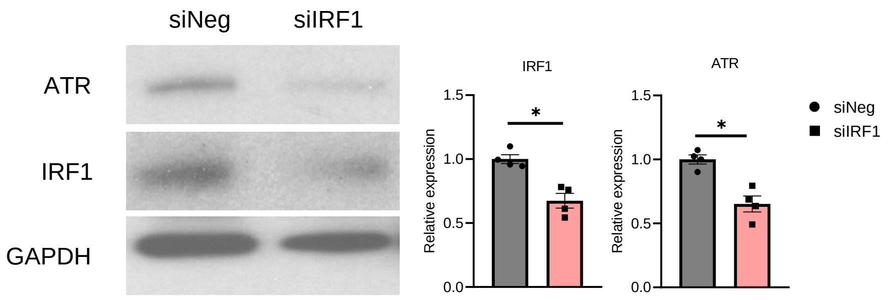

Fig. S15. Efficiency of IRF1 silencing. B cells were isolated from healthy individuals (n=4) using magnetic bead-based approach and were cultured ex vivo and transfected with siIRF1 or siNeg (scramble, control) for 42h, followed by IFNa and IL21/ CpGb/ sCD40L survival and mild proliferation stimuli exposure till day 4 where western blot analysis was performed with ATR and IRF1 antibodies. GAPDH blotting was also applied to confirm equal loading of each sample. Representative samples depicted. Results are presented as mean ± SEM. *p<0.05 (paired Student’s t test).

Figure S16

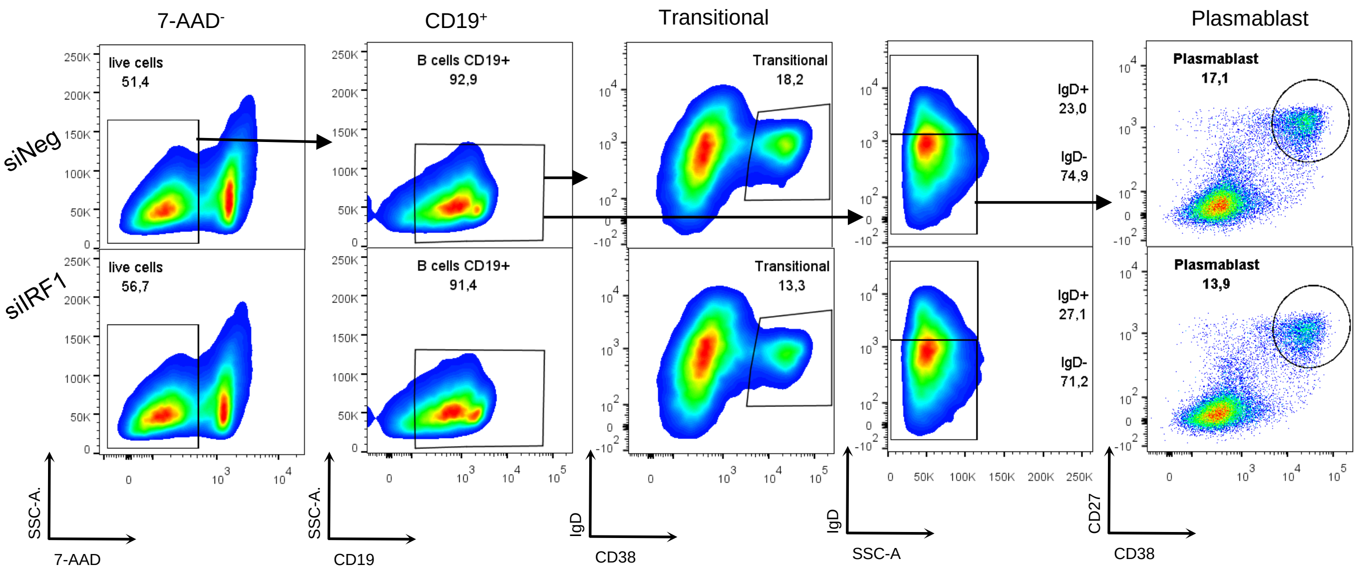

Fig. S16. IRF1 knockdown decreases formation of transitional and plasmablast in IFNα-treated B cells. The gating strategy on flow cytometry for assessing transitional ($CD19^{+}IgD^{dim}CD38^{+}$) and plasmablast ($CD19^{+}IgD^{-}CD27^{+}CD38^{+}$) B cells upon siIRF1 or siNeg (control) (Fig. 6).

Legends for Tables S1-S3 (separate files)

Table S1: Clinical and demographic characteristics of the subjects enrolled in the study. Age and sex are depicted for all individuals (SLE, AS, HC). For the patients with SLE, information on SLEDAI, PGA, organ involvement, disease status, disease duration and type of treatment at the time of sampling is also reported. AS: Ankylosing spondylitis; SLE: Systemic lupus erythematosus; HC: healthy control; SLEDAI: Systemic lupus erythematosus disease activity index; PGA: physician global assessment; F: female; M: male; LN: Lupus nephritis; NPSLE: Neuropsychiatric lupus; ACS: acute coronary syndrome; MTX: methotrexate; HCQ: hydroxychloroquine; AZA: Azathioprine; BEL: Belimumab; GCs: Glucocorticoids; *: samples used for the proteomic application.

Table S2: Proteomic enrichment analyses. Full lists of protein input data and enriched terms as revealed by IPA and STRING analyses.

Table S3: Antibodies used for flow cytometric, confocal microscopy, western blotting and ChIP assays. Target, fluorochrome, clone, vendor, catalog number, application and dilution of the antibodies are reported.
